# Standardized body condition scoring system for tropical farm animals (large ruminants, small ruminants, and equines)

**DOI:** 10.1007/s11250-025-04328-4

**Published:** 2025-03-07

**Authors:** Eric Vall, Mélanie Blanchard, Ollo Sib, Boris Cormary, Eliel González-García

**Affiliations:** 1 CIRAD, UMR SELMET, 34398 Montpellier, France; 2 SELMET, Univ Montpellier, CIRAD, INRAE, Institut Agro, Montpellier, France; 3 CIRAD, UMR SELMET, Dakar, Senegal; 4 CIRAD, UMR SELMET, Camagüëy, Cuba; 5 INRAE, UMR SELMET, Univ Montpellier, CIRAD, INRAE, Institut Agro, Montpellier, France

**Keywords:** Body condition assessment, Zebu, Cattle, Buffaloes, Camels, Sheep, Goats, Donkeys, Tropic

## Abstract

In tropical regions, subject to significant fluctuations in feedstuffs, the body condition score (BCS) is a relevant indicator for monitoring body reserves status of farm animals. However, the most used BCS grids comes from temperate conditions and still not adapted for tropical species, which limits adoption and usefulness. The current work presents for the first time an original, low-cost, standardized BCS assessment system, suited to a large spectrum of tropical farm animals (zebu and/or crossbred cattle, buffaloes, camel, sheep, goats, horses and donkeys). Based on a rigorous set of uniform criteria for practical, easy-to-use on field conditions, the animal model used to calibrate and validate each grid is the adult female (except the male for horses and donkeys). A six-point BCS grid system is proposed (i.e., from very emaciated -0- to overweighed -5- body conditions), based on visual interpretation of the back and right-lateral sides of each animal. The overall BCS assessment criteria is built on three major anatomical regions (hindquarters; thorax and abdomen; shoulder and neck), and ten related anatomical landmarks, which provide a unique consensual global interpretation of the back and right views. On-field tests of the proposed BCS grids for each animal species revealed acceptable reproducibility with regard to the most conventional, established gold-standards BCS methods (r^2^ = 0.67–0.99). The proposed harmonized BCS is relatively simple and must facilitate the adoption of regular, accurate BCS assessment by adequately trained operators linked to tropical animal production environments.

**Supplementary Information:**

The online version contains supplementary material available at 10.1007/s11250-025-04328-4.

## Introduction

The body condition score (BCS) is a widely used and cost-effective method for monitoring the body condition of farm animals, as it is linked to the energy balance and feeding systems. It indirectly reflects the overall welfare status of animals and the individual body reserves (i.e., lipid tissue and muscle mass), required for matching requirements of growth, milk production, work (e.g., animal traction) or reproduction. Numerous studies have highlighted the importance of BCS in maintaining the health and productivity of domestic farm species (for cattle (*Bos Taurus* and *Bos indicus*): Van Niekerk 1982; Nicholson and Sayers 1987; Roche et al. 2009; Béchir et al. 2010; for buffaloes: Tariq et al. 2013; Carvalho-Delfino et al. 2018; for camels: Faye et al. 2002; for sheep: Bocquier et al. 1999; Yilmaz et al. 2011; Kenyon et al. 2014; Soto-Barrientos et al. 2018; for goats: Hervieu et al. 1991; Cissé et al. 1992; Mendizabal et al. 2011; Battini et al. 2014; for horses: Westervelt et al. 1976; Dugdale et al. 2012; Banse and McFarlane 2014; for donkeys: Valle et al. 2017). Scoring and assessing BCS changes have become a strategic tool in both farm management and research scenarios. Consequently, this parameter has been extensively studied worldwide mainly in large and small ruminants (Roche et al. 2004; Bell et al. 2018) for cattle and sheep, respectively.

Thus, monitoring the animals body condition with the help of low-cost and non-invasive effective tools like BCS is highly relevant in the current context promoting farming system resilience and sustainability. This is particularly significant in tropical countries, which face marked forage seasonality and unpredictable fluctuations in the quantity and quality of feedstuffs available for animal feeding throughout the year, leading to underfeeding situations, especially on native rangelands. Furthermore, low-income tropical farming systems face challenges in being adequately equipped with common tools such as weighing scales. Other alternative methods (e.g., imaging techniques, ultrasonography), are also available in other latitudes and regularly used in precision livestock farming (for dairy cows: Ferguson et al. 2006; Schroder and Staufenbiel 2006; Halachmi et al. 2008; Bell et al. 2018; for dairy goats: Vieira et al. 2015). Different is the situation in the numerous smallholders or transhumant, nomad farmers found in tropical countries.

The most widely used BCS grids worldwide have been developed in temperate conditions (for dairy cattle: Croxton and Stollard 1976; Lowman et al. 1976; Buxton 1982; Edmonson et al. 1989; Ferguson et al. 1994; for sheep: Russel et al. 1969; for goats: Hervieu et al. 1991; Santucci et al. 1991; for horses: Henneke et al. 1983; Carrol and Huntington 1988). However, in tropical regions farm animals’ species and breeds present morphological characteristics very often different (e.g., frame, body size or hair coat). This is why many authors have proposed specific BCS grids, better adapted to tropical and warm areas breeds [for zebu (*Bos indicus*) cattle: Pullan 1978; Nicholson and Butterworth 1989; Ayala et al. 1992; Cissé 1995; Vall et al. 2002; Vall and Bayala 2004; for *Bos Taurus* cattle: Frantz 1988; Ayala et al. 1992; Van der Merve and Stewart 1995; Ezanno 2002; for buffaloes (*Bubalus bubalis)*: Ezenwa et al. 2009; Anitha et al. 2010; for camels (*Camelus dromedaries*): Faye et al. 2002; Iglesias et al. 2020; for sheep (*Ovis aries*): Richard 1997; for goats (*Capra hircus*): Poisot 1988; Honhold et al. 1989; Imadine 1991; Gosh et al. 2019; for horses* (Equus caballus)*: Diaw 2013; for donkeys (*Equus asinus*): Pearson and Ouassat 2000; Vall et al. 2001).

However, as shown in Table 1, all these BCS grids adapted to tropical breeds are heterogeneous in terms of 1) number of scoring levels (4 to 9); 2) views used to assign a BCS score (rear, right side, or other); 3) additional observations (e.g., palpation of lumbar areas for sheep and goats); 4) number of body landmarks considered (4 to 10). Such heterogeneity, even if better adapted to tropical conditions, reinforces the subjective nature of this practical body condition parameter, which has been pointed out by many authors (Nicholson and Sayers 1987; Bell et al. 2018). Furthermore, the access toto such adapted BCS grids becomes difficult, as most of them have not been published in peer review journals (e.g., Frantz 1988; Poisot 1988; Imadine 1991; Cissé 1995; Richard 1997; Ezanno 2002). Thus, the practical adoption and on-field uses still limited e.g., for regular monitoring of animals or to make comparisons between case studies using BCS grids based on different criteria. As a result, many researchers working in tropical conditions still using BCS assessment systems from temperate regions, which induce biases in their practical application and interpretation.

**Table 1:** BCS grids used for farm animal species reared in tropical and warm regions

Animal Specie	Breed (Country)	Level of scoring	Views used to assess			Body landmarks used to assess			
			Back view	Right side view	Other views	Pelvis and hip bones	Base of the tail	Pelvis cover	Thighs cover
Zebu	White Fulani (Nigeria)	6	×	×		×			
Zebu	*Bos indicus* in general	9	×	×		×	×	×	
Zebu	Gobra (Senegal)	6	×	×		×	×		
Zebu	White & Red, Fulani, Draft zebus (Cameroon)	6	×	×		×		×	×
Zebu	Sudanese (Burkina Faso)	6	×	×		×	×	×	×
Zebu	*Bos indicus* & Holstein (Mexico)	9				×	×	×	
Cattle	Ndama (Sub-Saharan Africa)	6	×	×	Loin area	×	×	×	
Cattle	Dairy cows (South Africa)	5	×		Loin area	×	×		
Cattle	Creole cow (Guadeloupe)	5	×		Left side	×	×		
Cattle	*Bos taurus* & Holstein cattle (Mexico)	9				×	×	×	
Buffalo	Murra buffaloes	5				×	×	×	
Buffalo	African buffaloes	5	×			×	×		
Camel	Maghrebi camel (Morocco)	6	×	×		×	×	×	
Camel	Canarian camel breed (Spain)	6	×		Left side	×	×	×	
Sheep	Fulani sheep (Sahel)	6			Lumber area	×			
Goat	Goat of sub-Saharan Africa (Tchad)	4			Lumbar and sternal areas	×		×	
Goat	Goat (India)				Left side	×		×	
Goat	Creole goat (Guadeloupe)	5				×			
Goat	Small East African goats (Zimbabwe)	7			Lumbar area				
Horse	Barbe horse (Senegal)	5	×	×		×	×	×	
Donkey	Drat donkeys (Cameroon)	4	×	×		×	×	×	
Donkey	Draft donkeys (Morocco)	9	×		Left side	×		×	
Animal Specie	Body landmarks used to assess							References	
	Lumbar vertebrae	Hollow side	Backline	Ribs	Shoulder	Neckline	Others landmarks		
Zebu	×		×					Pullan (1978)	
Zebu	×	×	×	×	×			Nicholson and Butterworth (1989)	
Zebu	×	×	×	×			Hump	Cissé (1995)	
Zebu	×	×	×	×			Hump	Vall et al. (2002)	
Zebu	×	×	×	×				Vall and Bayala (2004)	
Zebu								Ayala et al. (1992)	
Cattle	×			×				Ezanno (2002)	
Cattle	×							Van der Merve and Stewart (1995)	
Cattle	×	×						Frantz (1988)	
Cattle								Ayala et al. (1992)	
Buffalo	×		×	×				Anitha et al. (2010)	
Buffalo	×		×	×			Coat	Ezenwa et al. (2009)	
Camel	×	×	×	×	×		Palpation	Faye et al. (2002)	
Camel	×	×	×	×			Hump	Iglesias et al. (2020)	
Sheep	×		×				Palpation of lumbar area	Richard (1997)	
Goat	×		×	×			Palpation sternal and lumbar areas	Imadine (1991)	
Goat	×	×			×		Sternum cover	Gosh et al. (2019)	
Goat	×		×	×			Palpation of sternum and lumbar area	Poisot (1988)	
Goat	×	×	×				Palpation	Honhold et al. (1989)	
Horse	×		×	×	×	×		Diaw (2013)	
Donkey	×	×	×	×				Vall et al. (2001)	
Donkey	×		×	×	×	×	Muscle development	Pearson and Ouassat (2000)	

To address such issue of lacking a homogenous metric for BCS, our research unit (UMR SELMET; https://umr-selmet.cirad.fr/) has been working on developing a harmonized system, adapted to a large spectrum of farm animals species and breeds reared and expanded in different tropical settings. To carry out this work, we have been collaborating since 2019 with researchers and various stakeholders in the livestock sector (farmers, agricultural advisors, and veterinarians) from different regions of sub-Saharan Africa (Burkina Faso, Ivory Coast, and Senegal), Vietnam and Cuba.

The work presented here provides, for the first time, a standardized BCS assessment system adapted to a wide range of tropical farm animals (zebu and/or crossed cattle, buffaloes, camels, sheep, goats, horses and donkeys), based on a rigorous set of uniform criteria for practical and easy-to-use field conditions.

## Methodology: proposal of an original, multispecific, standardized BCS assessment system

### Animal species targeted

The work was carried out in several tropical sites of Western Africa (Burkina Faso, Ivory Coast, and Senegal), South-East Asia (Vietnam) and Latin America (Cuba). The following specific tropical breeds and/or animal species (*Bos indicus* –zebu- and *Bos Taurus* cattle, buffaloes, camels, sheep, goats, horses and donkeys) were targeted, for developing specific BCS grids based on the same principles:

- *Bos indicus* –zebu- and *Bos taurus* cattle:
  - Sudanese zebu (*Bos indicus*) of Sahel and Savannah areas of West Africa
  - Ndama cattle (*Bos taurus*) in Ivory Coast
  - Crossbred *Bos indicus* × *Bos taurus*: Dairy cattle (Cuba) and Yellow cattle (Vietnam)
- Buffaloes (*Bubalus bubalis*) in Vietnam
- Camels (*Camelus dromedaries*) in Sahel and Saharan areas of West Africa
- Small ruminants (of Sahel and Savannah areas of West Africa):
  - *Djalonké* sheep (*Ovis aries*)
  - Goats (*Capra hircus*)
- Equines:
  - Barbe horses (*Equus caballus*) in Senegal
  - Donkeys (*Equus asinus*) of Sahel and Savannah areas of West Africa

### The principles of the standardized BCS system

In the proposed BCS system, the animal model is the adult female. In *Bos indicus* –zebu- and *Bos taurus* cattle, buffaloes, camel, sheep and goat herds, females are the most numerous and important component for the reproduction, playing a key role in keeping a sustainable longitudinal functioning of the flock or herd in a long timespan path. The BCS of the adult female is the best indicator of the quality of the established feeding systems, the inherent energy status of individuals and the overall management of the farm. For example, if a large proportion of emaciated females is present in the herd, it indicates a poor feeding system and/or nutritional management on the farm. Contrary to other species, for equids (donkeys and horses), the animal model chosen was the adult male, considering their often use for animal traction, as draft animals, for land works, cropping and transport.

The proposed BCS system is based on six assessment levels, ranging from 0 (emaciated) to 5 (overweighed). The reason for choosing an intermedium six level scoring is two-fold: firstly, it corresponds to what most often is proposed in other grids (Table 1); and secondly, too many levels add difficulty in practice (thus requiring more training and affecting reproducibility of the operators) whereas too few levels affect the accuracy in the assessment.

**Fig. 1:**
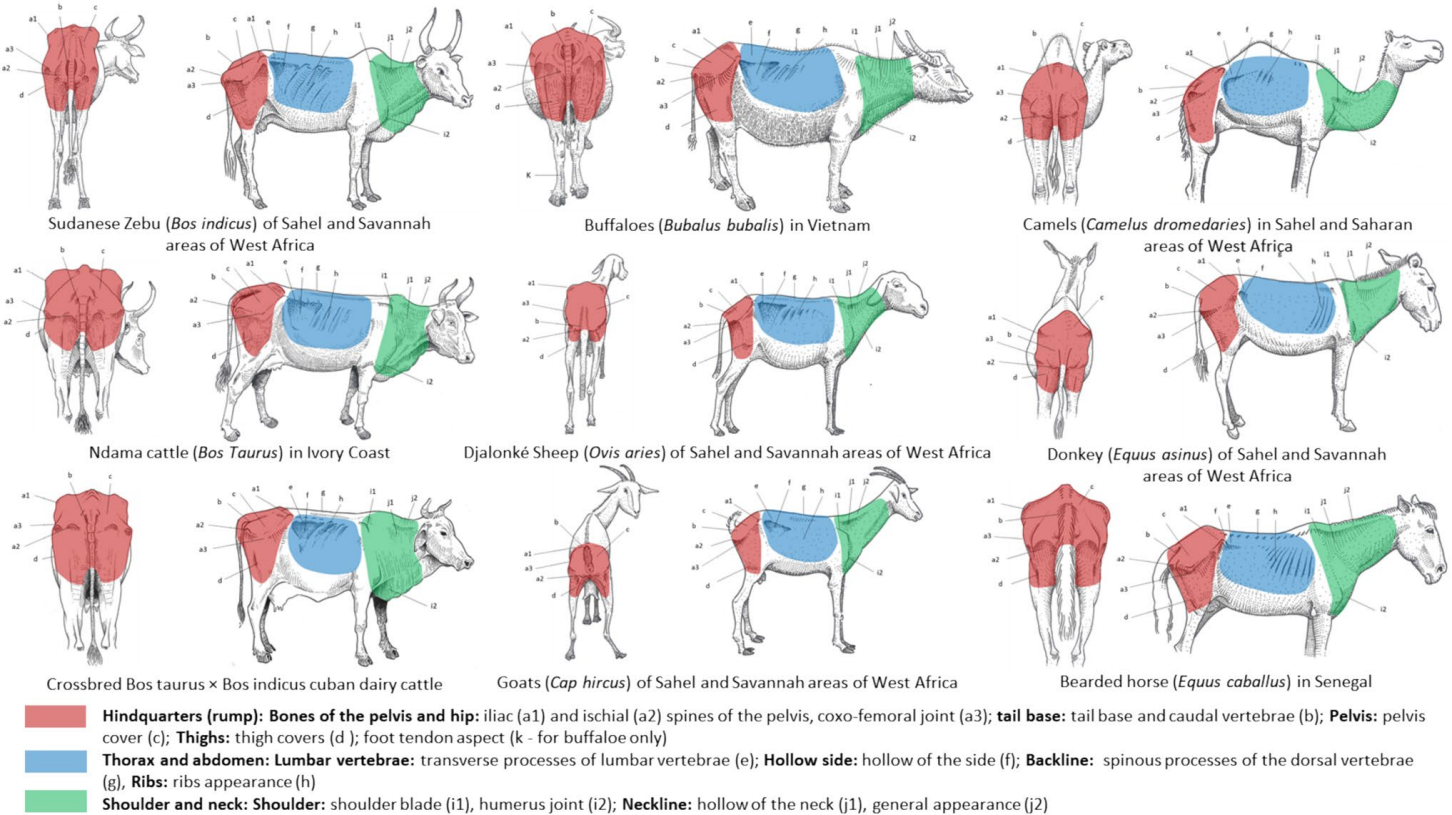
Anatomical regions and body landmarks considered in the proposed standardized body condition scoring system

The proposed BCS system is based on visual interpretation of the back and right sides of each animal to establish an overall assessment criterion across three anatomical regions (i.e., the hindquarters or rump, the thorax and abdomen, and the shoulders and neck; Fig. 1).

Then, the scoring is based on the visual observation of the following body landmarks:

1. the bones of the pelvis and hip [iliac (a1) and ischial (a2) spines of the pelvis, coxofemoral joint (a3)];
2. the base of the tail [caudal strait and caudal vertebrae (b)],
3. the pelvis (corresponding to the point c);
4. the thighs (corresponding to the point d);
5. the lumbar vertebrae (transverse apophyses of the lumbar vertebrae (e);
6. the hollow side (corresponding to the point f);
7. the back line [spinous apophyses of the dorsal vertebrae (g)];
8. the ribs (corresponding to the point h);
9. the shoulder [scapula (i1), humerus joint (i2)]
10. the neckline [hollow of the neck (j1) and general appearance (j2)]

A first assessment is carried out for each anatomical region or landmark. A BCS score is then assigned for the back and right-side views, respectively. The average of the two scores provides the overall individual's BCS assessment, which is based on a global interpretation of all body landmarks. According to the average BCS score assigned, individual animals are classified as ‘Very emaciated, skeletal animal’ (Score 0), ‘Very thin animal’ (Score 1), ‘Skinny, lean animal’ (Score 2), ‘Animal with good body condition’ (Score 3), ‘Animal with excellent body condition’ (Score 4), ‘Fat and smooth, overweighed animal’ (Score 5). The same criteria were used to validate the system for all the animal species involved in this work (Fig. 1).

### The BCS grids conception and design (source of drawings)

For the BCS score grids, we opted for using hand-made representative drawings rather than pictures taken with cameras. This is because it is not easy to get pictures clearly presenting the details of the anatomical points in the different positions of the animal, as was done by most authors proposing previous BCS grid systems (Poisot 1988; Honhold et al. 1989; Cissé 1995; Richard 1997; Ezanno 2002; Faye et al. 2002; Vall et al. 2001; Vall and Bayala 2004; Anitha et al. 2010; Gosh et al., 2019; Diaw 2013; Iglesias et al. 2020). Even if, some grids are presented with photos (Pullan 1978; Nicholson and Butterworth 1989; Pearson and Ouassat, 2000). Nevertheless, to develop the drawings we used pictures taken to live animals, representing both views (back and right sides, photos taken at 3 m each), and the range of body condition (from emaciated to overweighed), under field conditions, there where animals from the different species and breeds involved in this study were present (Table 2).

**Table 2:** Number of pictures, taken at 3 m of distance, and used to create the BCS grids according to the two complementary views of a given animal with a given body condition

Animal species	Back view						Right side view					
	0	1	2	3	4	5	0	1	2	3	4	5
Sudanese zebu	1	1	2	4	1	1	1	1	3	3	1	1
Ndama cattle	1	2	4	1	1	1	1	3	3	1	1	1
Crossbred dairy cattle (Cuba)	1	3	2	3	4	2	1	4	2	2	4	2
Crossbred Yellow cattle (Vietnam)	1	2	4	5	3	3	1	2	4	4	5	2
Buffaloes	1	2	2	1	1	1	1	1	2	1	1	2
Camels	0	1	2	2	3	2	1	2	3	1	2	1
Djalonké Sheep	1	3	3	1	1	1	1	2	1	3	1	2
Goats	1	2	3	3	1	0	1	1	3	3	2	1
Donkeys	1	1	3	2	2	1	0	1	1	2	5	1
Horses	1	2	2	4	1	0	1	1	2	4	1	1

The drawings represent a typical animal for each of the different BCS levels and from two perspectives angles (right side view, back view). In agreement with that, each animal was photographed from the two perspectives, at approximately a 3 m distance, combining prudence for avoiding disturbance to the animal while warranting the quality of the picture. For this purpose, the animals were controlled, kept in standing position in the free stalls or lot areas and/or restrained in headlocks at the feeders.

From the drawing of a typical animal presenting an average soring (BCS = 2) in both views (back view, right side view), and pair of photos corresponding to each score level, the appearance of the typical animal (and of all the corresponding body landmarks) was developed to obtain 6 drawings of a typical animal in back and side perspective corresponding to each score level. In this paper, all draws were hand-made based on representative pictures taken on field, by the first and corresponding author of this work i.e. Eric Vall.

### Validation of the BCS grids by experts and local stakeholders

To validate the constructed BCS grids we organized workshops with livestock experts from each locality involved in the project:

- Ouagadougou workshop (Burkina Faso) in 2020 with around 22 experts from the countries of the Inter-State Committee for Drought Control in the Sahel (CILSS: Burkina Faso, Senegal, Mauritania, Niger, and Chad), for the validation of the BCS Sudanese zebu, camels, Djalonké sheep, goats, and donkeys’ grids;
- Dien Bien Phu workshop (Vietnam) in 2021 with around 20 experts for the validation of the BCS buffaloes and Yellow cattle grids;
- Bouaké workshop (Ivory Coast) in 2022 with around 18 experts for the validation of the BCS Ndama cattle grid;
- Camagüey (Cuba) in December 2024 with around 21 participants coming from different fields or stakeholders related to livestock production in the region; they validated the BCS grid conceived for crossed *Bos indicus* × *Bos taurus* dual purpose cattle

Initially, the workshops participants were trained in the principles of BCS assessment and how to use the BCS grid projects to be validated. Then, they practiced such principles using BCS grid projects during supervised scoring sessions on pictures, then during a practical scoring work for assessing live animals on local markets or directly on farms.

#### Evaluating the reproducibility of the BCS assessment (“scoring”)

Thereafter, the scoring data collected during the tutorials and practical work made it possible to assess the reproducibility of the scoring for each BCS grid. According to Agabriel et al. (1986), the reproducibility of the BCS is defined as the difference observed between the scores assigned to the same animal by different operators or scorers using the same system. To assess it in a simple analysis of variance model, reproducibility is defined by the correlation between the score given by one (trained, expert) judge, and those given by other judges during the same test. The Table 3 presents the number of scorers, the sources (pictures, living animals), and the number of views used to assess the reproducibility of the proposed BCS systems.

**Table 3:** Number of scorers, sources (pictures, living animals), and number of views used to assess the reproducibility of the proposed BCS systems

Animal species	Number of scorers	Pictures (P) Living animal (LA)	Rigth-side view (RSW)	Back view (BW)	Average RSD and BW
Sudanese zebu	22	P	10	10	×
	21	LA	12	12	12
Ndama cattle	18	P	10	10	×
	14	LA	14	14	14
Crossbred Cuban dairy cattle	21	P	20	20	20
	20	LA	18	18	18
Yellow cattle	20	P	10	8	×
	17	LA	3	3	3
Buffaloes	20	P	10	10	×
	17	L	3	3	3
Camel	22	P	10	10	×
Djalonké sheep	22	P	10	10	×
	21	LA	12	12	12
Goats	22	P	10	10	×
	21	LA	12	12	12
Donkeys	22	P	10	10	×

Finally, based on the lessons learned from the practical works made on the field, and the results of the reproducibility assessment sessions for each BCS grid, the participants proposed some modifications to be considered to improve the BCS grids for the description of body landmarks, before validating the final versions of the BCS grids.

#### Evaluating the scoring precision according to the grid level

For each animal and from the same angle or perspective (back or lateral views), we analysed the variance of the score attributed by a group of operators (“scorers”) in order to evaluate the accuracy of the BCS assessment (scoring) according to the level (0, 1, 2, 3, 4 or 5). Then, we calculated the mean of the performed BCS assessment for each animal and perspective, which was considered to be the reference BCS value (BCSref). For each animal species, the BCS of the scorers attached to the same BCSref were grouped in order to analyse the variance among the scorers. Finally, the 6790 BCS assessment collected throughout all workshops were retained and distributed according to the animal species and to the BCSref (Table 4).

**Table 4:** Numbers of BCS assessments used (across different countries) to analyse the accuracy of the standardized BCS system according to the animal species and the reference body condition scoring level (0 à 5)

Animal species	BCSref_0	BCSref_1	BCSref_2	BCSref_3	BCSref_4	BCSref_5	Total per species
Sudanese zebu	63	109	194	216	234	128	944
Ndama cattle	26	40	141	251	82	0	540
Crossbred Cuban dairy cattle	63	84	474	470	389	80	1560
Yellow cattle	0	63	181	139	97	0	480
Buffaloes	0	0	202	101	198	21	522
Camel	22	22	110	88	110	88	440
Djalonké sheep	0	152	215	256	237	84	944
Goats	0	44	274	232	370	0	920
Donkeys	0	44	88	132	154	22	440
Total per BCSref	174	558	1879	1885	1871	423	6790

## Results

### Standardized morphological regions and anatomical points

The Fig. 1 represents the anatomical regions and body landmarks considered in the proposed standardized BCS system for all the animal species involved in this study.

### Sudanese zebu

**Fig. 2:**
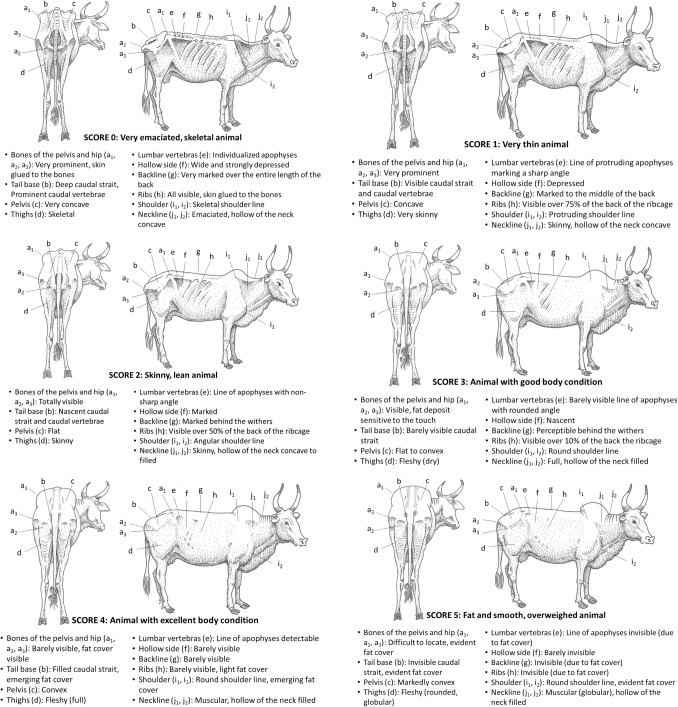
BCS grid for Sudanese zebu

The Fig. 2 presents the BCS grid for Sudanese zebu (Vall 2025f).

### Ndama cattle

**Fig. 3:**
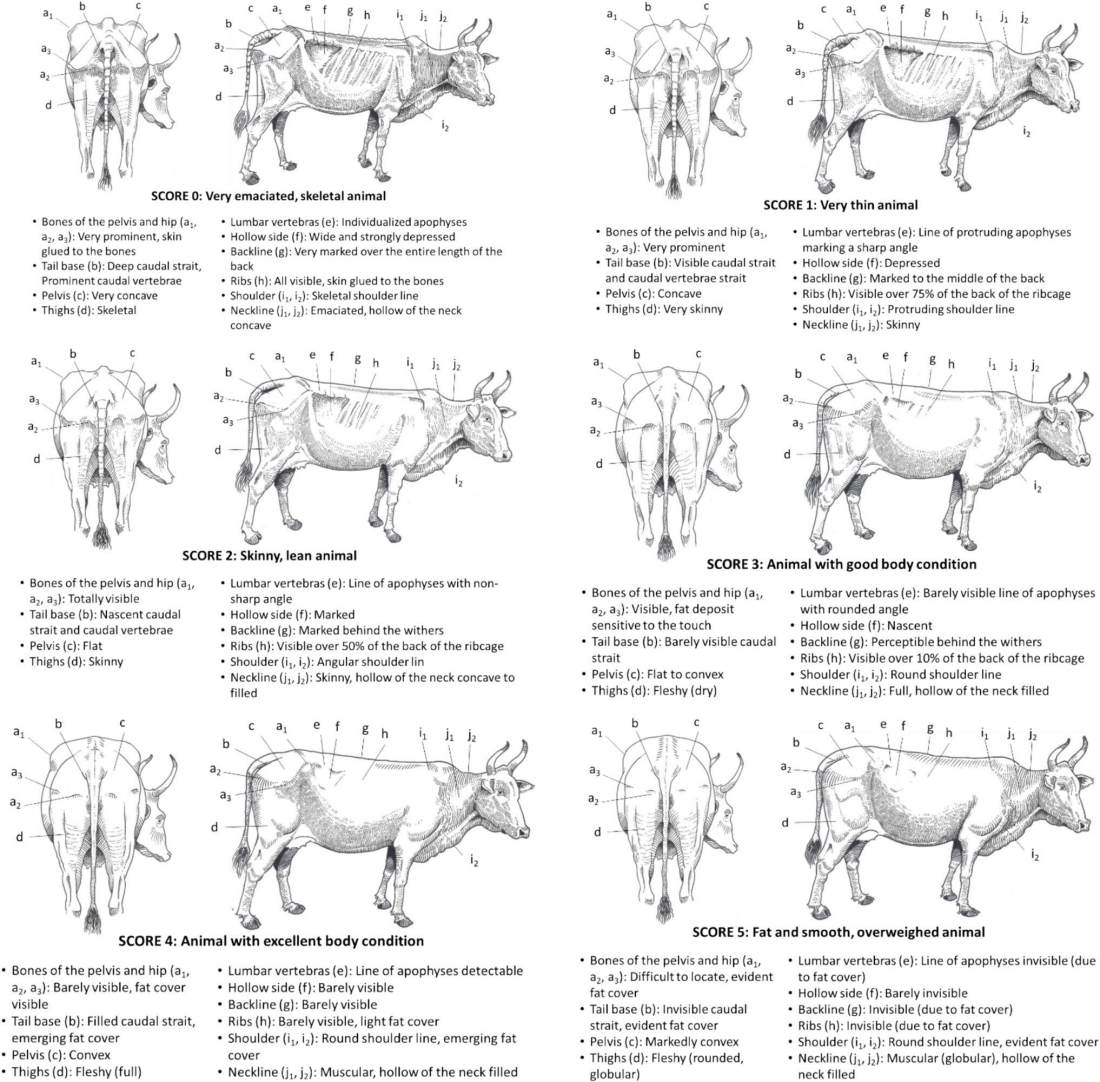
BCS grid for Ndama cattle

The Fig. 3 presents the BCS grid for Ndama cattle (Sib et al. 2025).

### Crossbred *Bos Taurus* × *Bos indicus* cattle

**Fig. 4:**
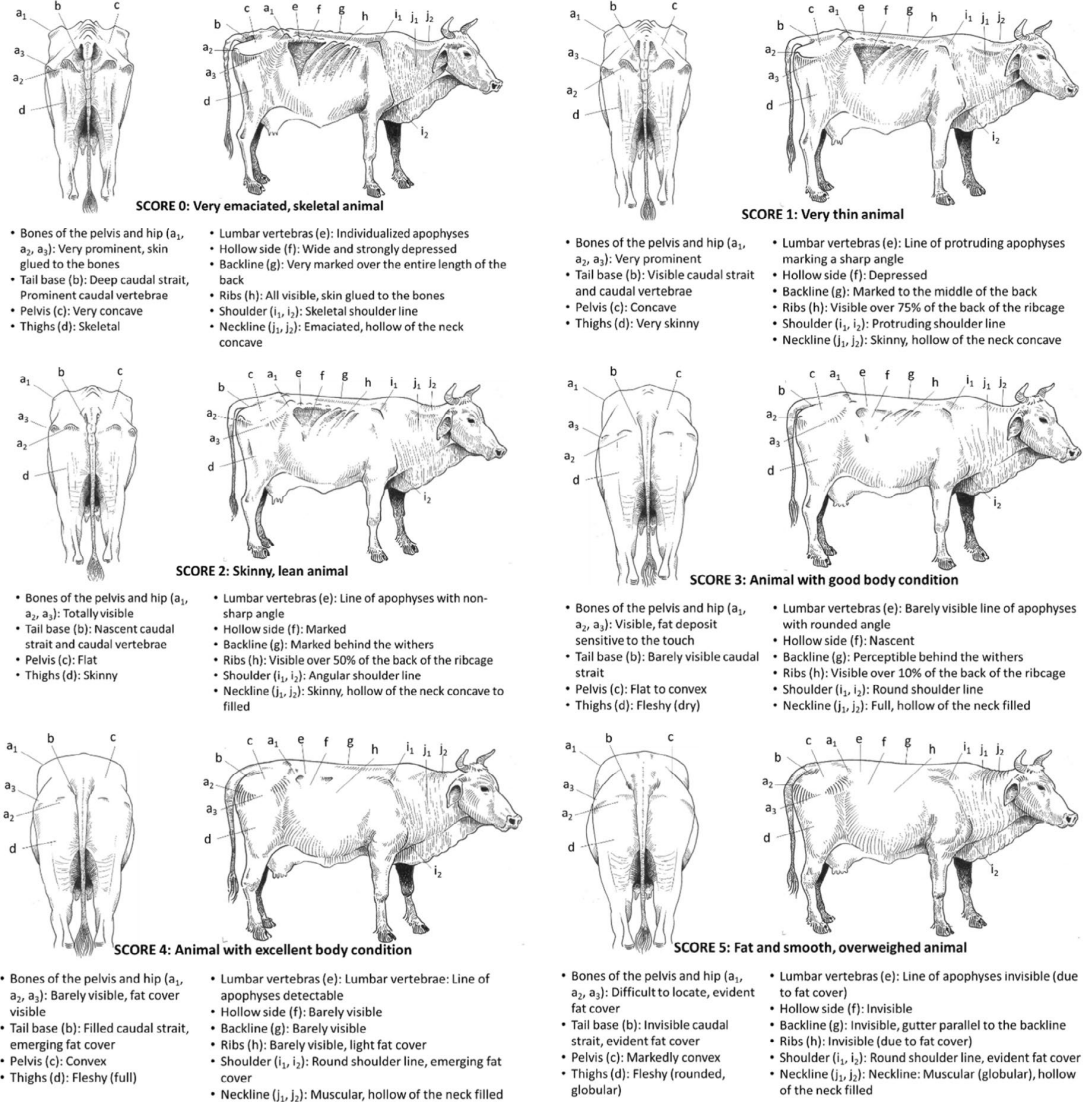
BCS grid for crossbred *Bos Taurus* × *Bos indicus* (Cuban dairy cattle)

The Fig. 4 presents the BCS grid for crossbred Cuban dairy cattle (Comary et al. 2025). The similar BCS grid for Yellow cattle (Vietnam) is not presented in this paper (Blanchard et al. 2025a).

### Buffaloes

**Fig. 5:**
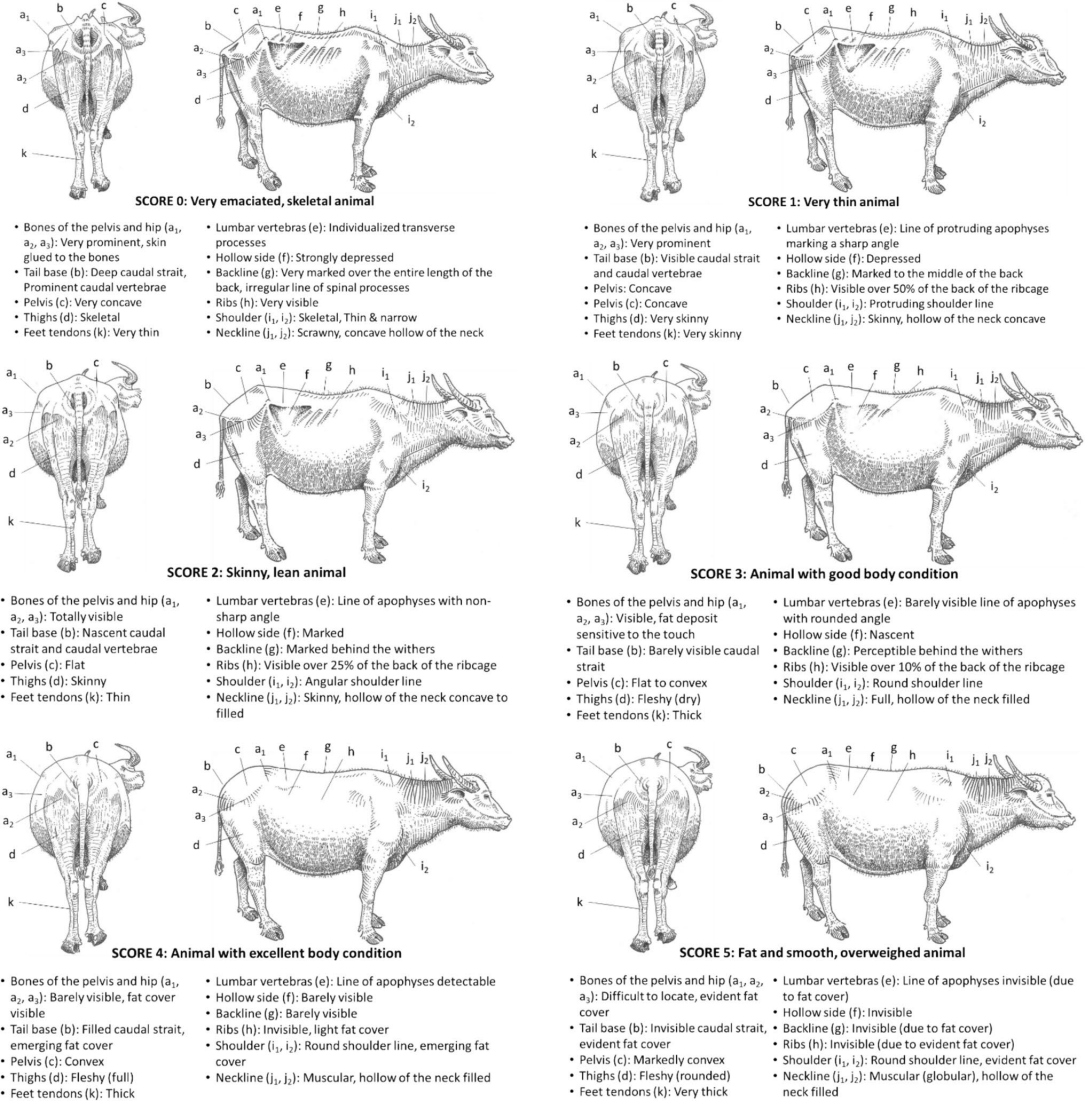
BCS grid for buffaloes

The Fig. 5 presents the BCS grid for buffaloes (Blanchard et al. 2025b)

### Camels

**Fig. 6:**
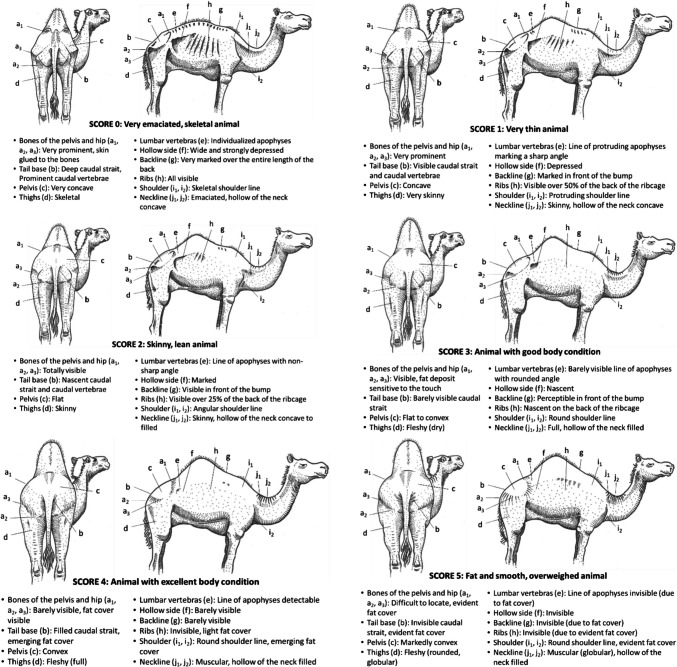
BCS grid for camel

The Fig. 6 presents the BCS grid for camels (Vall 2025b).

### *Djalonké* sheep

**Fig. 7:**
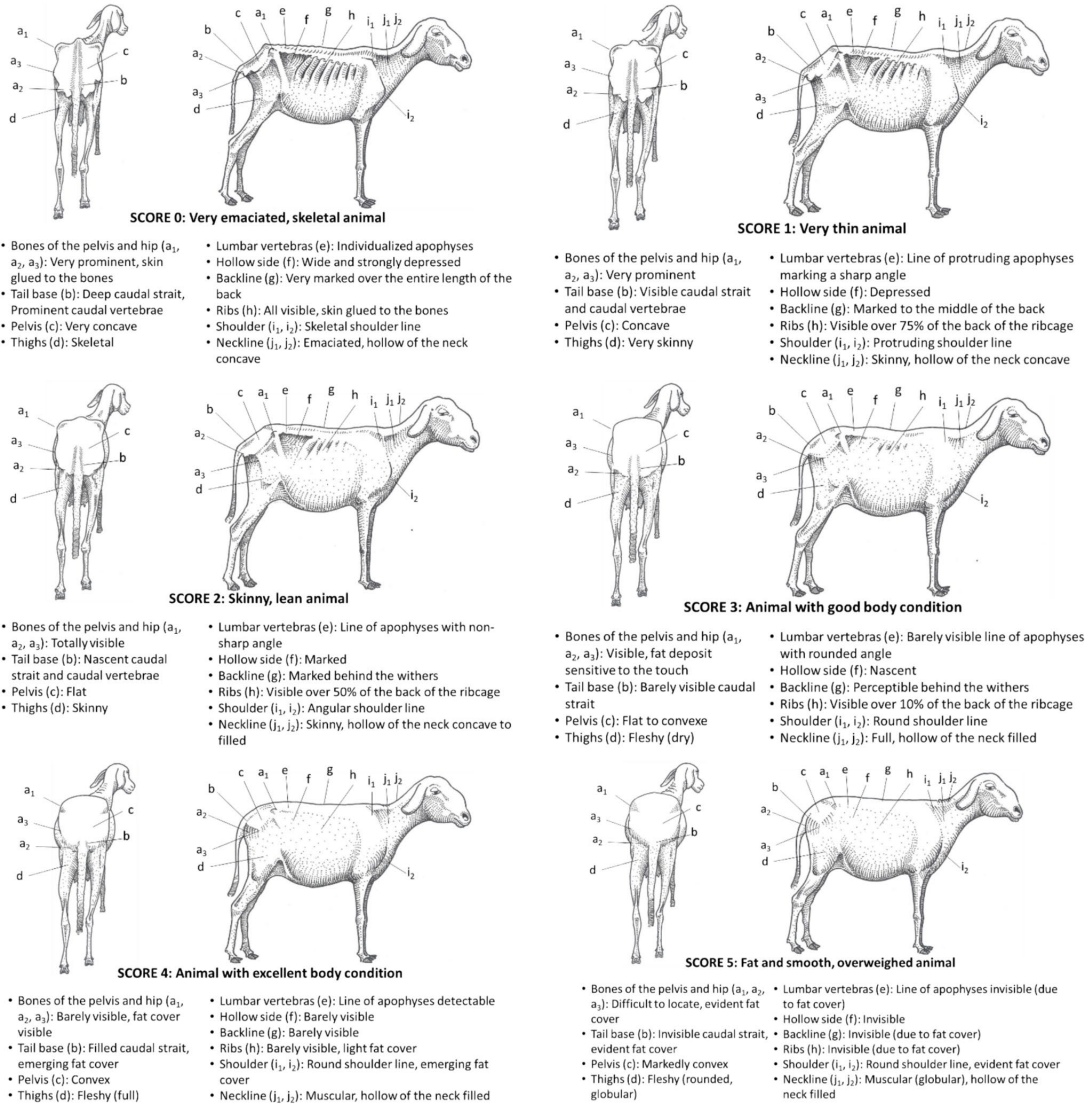
BCS grid for Djalonké sheep

The Fig. 7 presents the BCS grid for *Djalonké* sheep (Vall 2025e).

### Goats

**Fig. 8:**
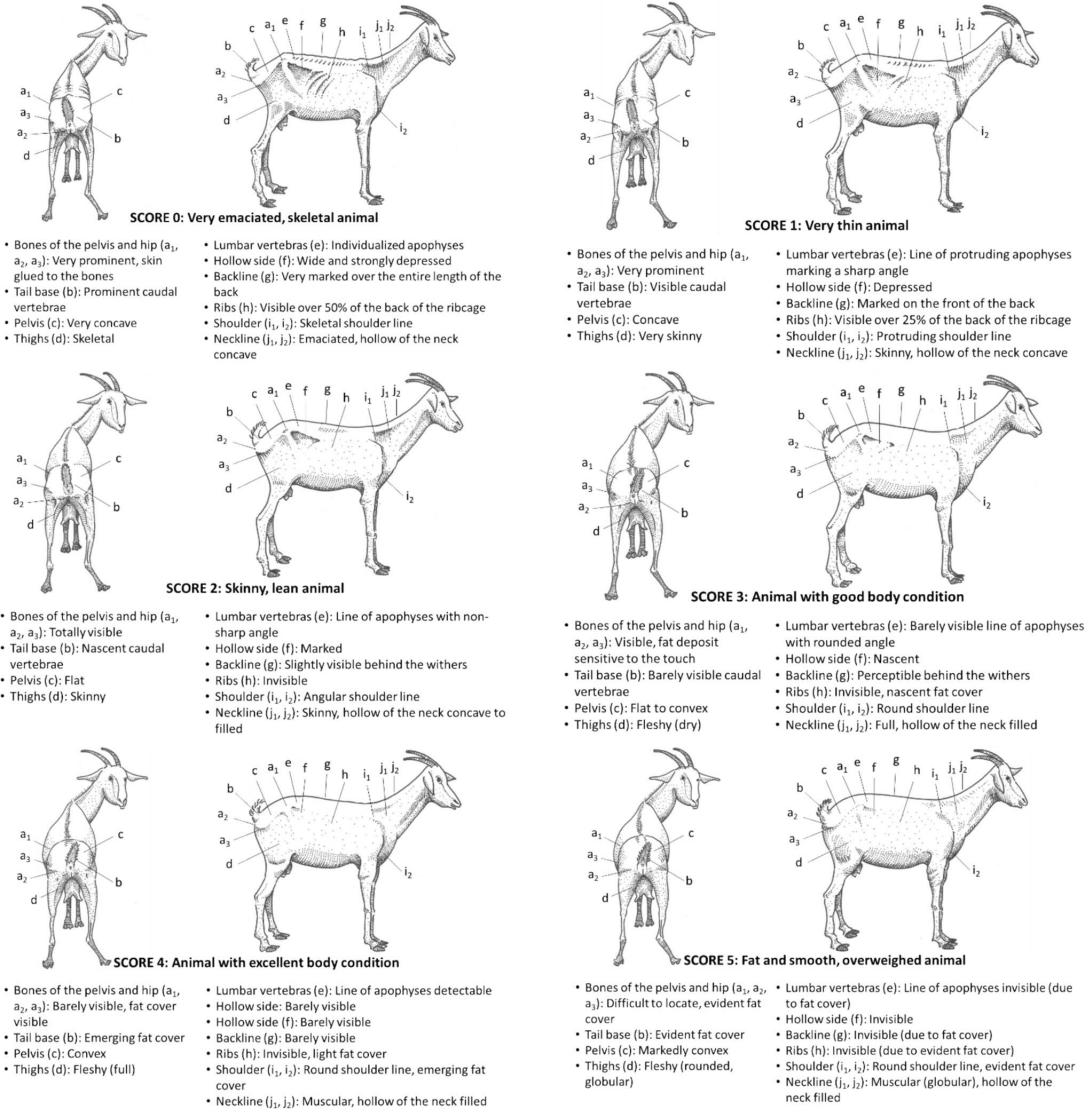
BCS grid for goat

The Fig. 8 presents the BCS grid for goats (Vall 2025d).

### Barbe horses

**Fig. 9:**
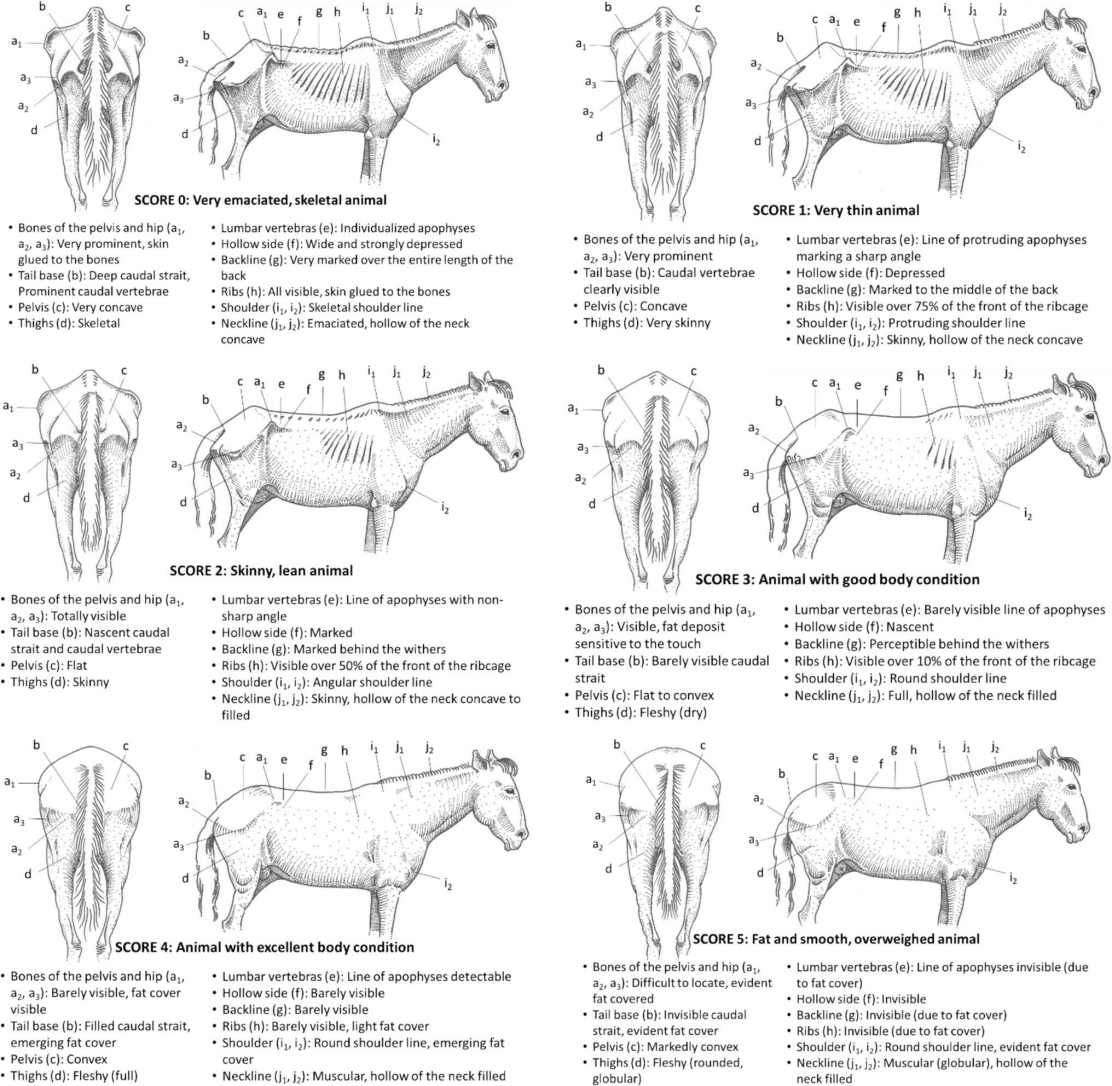
BCS grid for Barbe horse

The Fig. 9 presents the BCS grid for *Barbe* horse (Vall 2025a).

### Donkeys

**Fig. 10:**
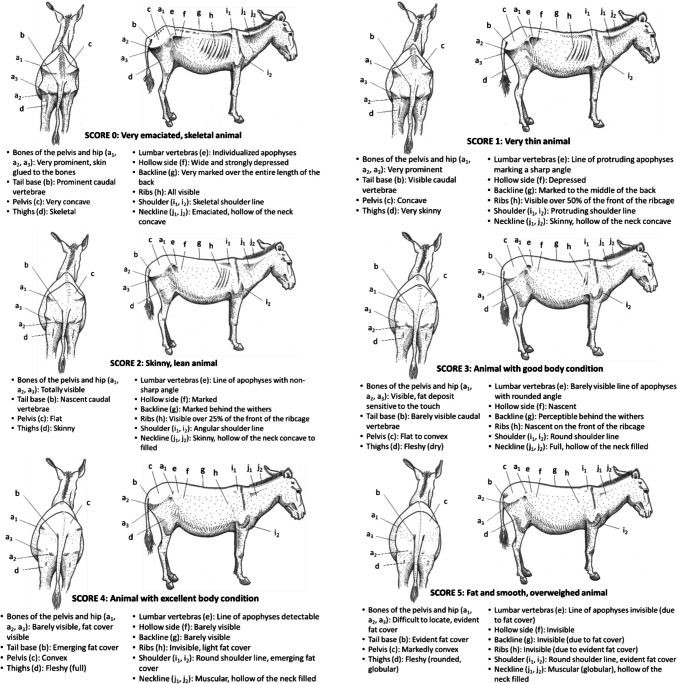
BCS grid for donkeys

The Fig. 10 presents the BCS grid for donkeys (Vall 2025c).

#### Reproducibility of the standardized BCS grids for tropical animals

The reproducibility assessment results presented in Table 5 show that: 1) Reproducibility (between 0.67 and 0.99) is acceptable for all animal species; 2) Reproducibility improves with the training of the operator charged of the scoring (the correlation coefficients of scoring on live animals carried out after photo scoring are higher); 3) The integration of the two scoring (right side view and back view) improves reproducibility.

**Table 5:** Reproducibility of BCS assessment

Animal species	Sources (*)	Number of Scorers	Views (**)	Pr > P α	Correlation (%)
Sudanese zebu	P	22	RSV	0.724	0.83
	P	22	BV	0.999	0.83
	LA	21	RSV	1.000	0.97
	LA	21	BV	1.000	0.97
	LA	21	AV	1.000	0.99
Ndama cattle	P	18	RSV	0.811	0.74
	P	18	BV	0.688	0.81
	LA	14	RSV	0.537	0.72
	LA	14	BV	0.146	0.68
	LA	14	AV	0.676	0.76
Crossbred Cuban dairy cattle	P	21	RSV	0.449	0.835
	P	21	BV	0.374	0.833
	P	21	AV	0.428	0.870
	LA	20	RSV	0.687	0.835
	LA	20	BV	0.914	0.831
	LA	20	AV	0.945	0.870
Yellow cattle	P	20	RSV	0.995	0.78
	P	20	BV	0.995	0.73
	LA	17	RSV	0.999	0.93
	LA	17	BV	0.987	0.90
	LA	17	AV	0.998	0.93
Buffaloes	P	20	RSV	0.992	0.69
	P	20	BV	0.788	0.77
	LA	17	RSV	1.000	0.87
	LA	17	BV	0.996	0.89
	LA	17	RSV	1.000	0.93
Camel	P	22	RSV	0.999	0.88
	P	22	BV	0.380	0.70
Djalonké sheep	P	22	RSV	0.914	0.67
	P	22	BV	0.960	0.76
	LA	21	RSV	1.000	0.89
	LA	21	BV	1.000	0.92
	LA	21	AV	1.000	0.95
Goats	P	22	RSV	1.000	0.85
	P	22	BV	0.870	0.84
	LA	21	RSV	1.000	0.86
	LA	21	BV	1.000	0.85
	LA	21	AV	1.000	0.90
Donkeys	P	22	RSV	0.445	0.72
	P	22	BV	0.467	0.82

Legend: (*) P: pictures; LA: living animals; (**) RSV: Right-side view; BV: Back view; AV: Average

#### Variance among operators of the BCS assessment (“scoring”) according to the BCS level of reference

**Fig. 11:**
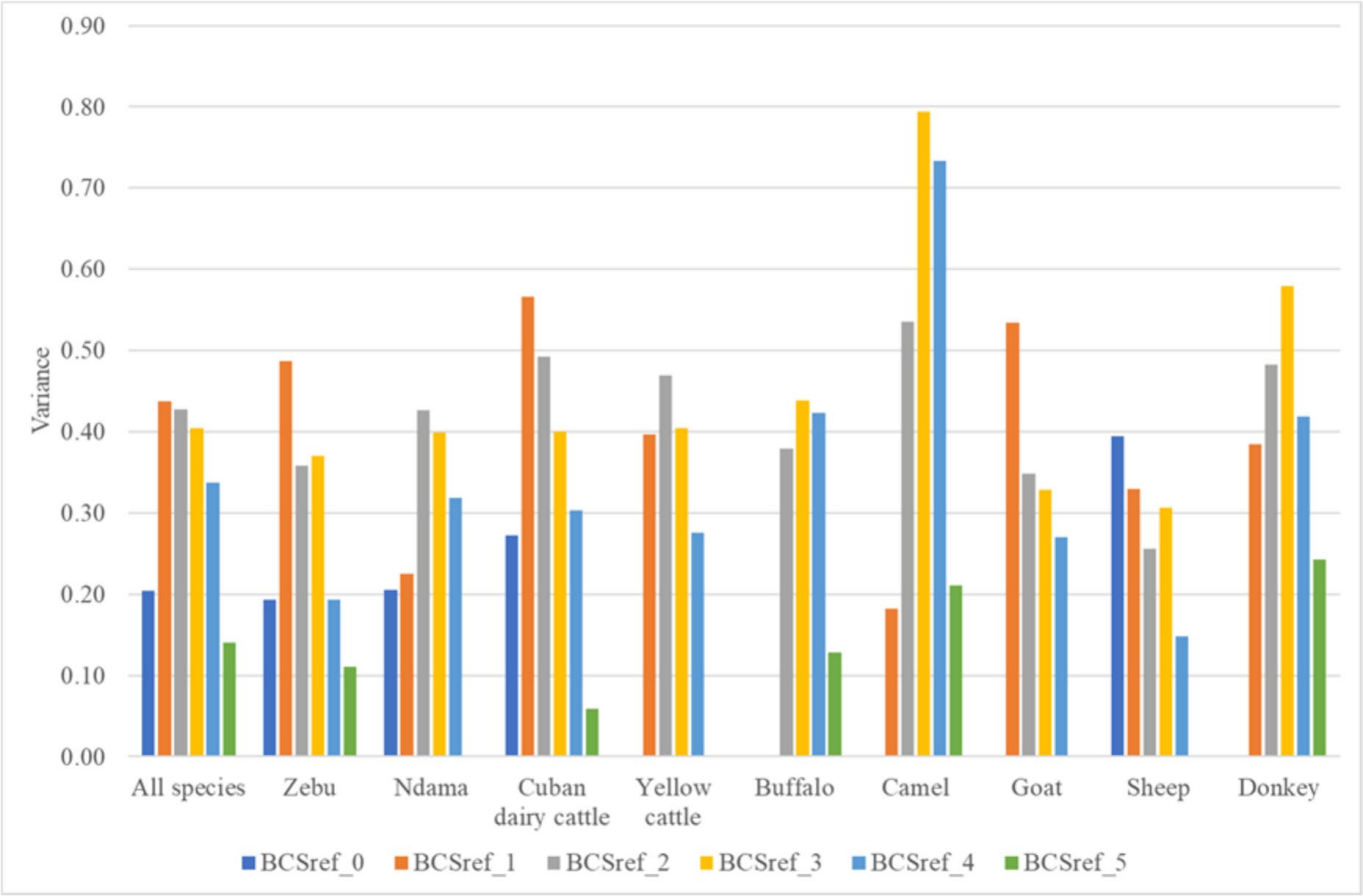
Variance in the BCS scores attributed per level in the BCS of reference for each grid (BCS ref: 0, 1, 2, 3, 4, 5), according to the animal specie

The Fig. 11 present, for all animal species, the variance of the BCS scores attributed by the operators, in function of the BCS of reference (BCSref: 0, 1, 2, 3, 4, 5). In most of the case, results show lower variance (i.e., more agreement among scorers in attributing a BCS value to a given animal from a given perspective) in the extreme levels of the grid (0 et 4 et 5) and, conversely, more the animal tend to be in the middle of the scale (BCS scores 1, 2, 3) more variance is observed among the scorers. This result highlights the importance of proposing some solutions (i.e., “tips” to avoid confusion and add clarity) to improve BCS assessment at these points of the grids.

**Fig. 12:**
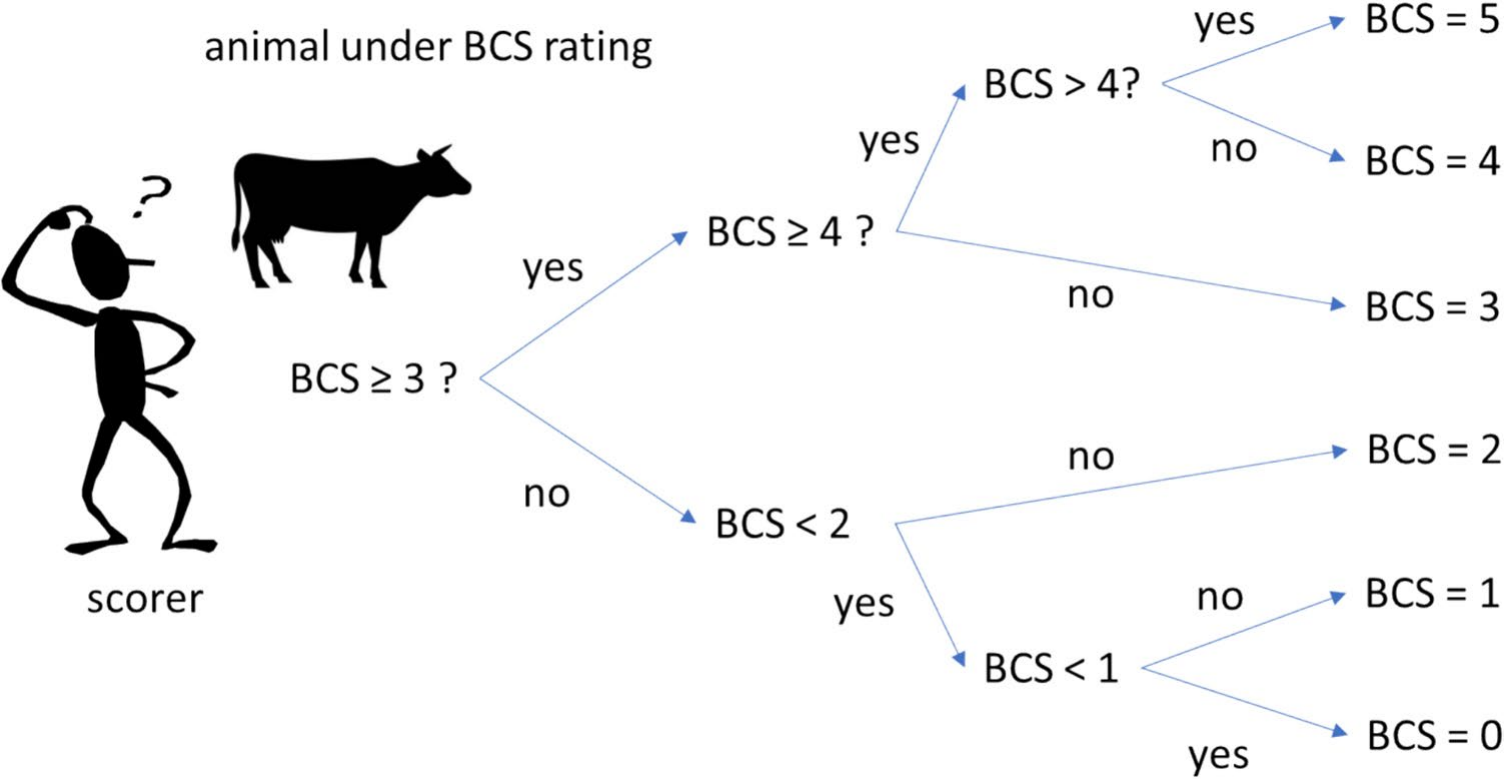
Decisional tree helping to attribute a body condition score (BCS) in the more precise and simple manner

To overcome confusion in the middle levels of the grids, we propose to follow in a methodical manner the decisional tree presented in the Fig. 12. A chain of three successive questions may be comprised, which may substantially improve the precision of the attributed score. The chain of this decisional tree must be then interpreted as follows:

- First question: is the BCS of this animal equal or higher to 3 -the mid of the grid-? (BCS ≥ 3?; Yes or Not). Answering this first question may be determinant as a first step as it will lead to discriminate the score in two opposite overall trends
- Second question, if the answer is Yes, i.e., the animal been assessed would be in a positive body condition or, by the contrary, in negative (if the answer is Not)

Third question, is then to apply the more precise “tips” leading to the specific extremes, for attributing the good score at the 0, 1, 4 and 5 levels above.

## Discussion and conclusion

The study presented in this paper proposes for the first time in a scientific (web indexed) journal a set of standardized BCS grids for farm animal species, reared in the tropics (zebu and/or crossed cattle, buffaloes, camels, sheep, goats, horses and donkeys). These grids are harmonized and based on a set of common principles, including the animal model chosen (adult females, except for equines), six scoring levels ranging from very emaciated (0) to overweight animals (5), two views (back and right-side), observation of three anatomical regions (hindquarters; thorax and abdomen; shoulder and neck), and ten body landmarks (Fig. 1).

This is a novel approach, as such harmonization in a large spectrum of farm animal species had not been previously attempted in either temperate or tropical latitudes (for zebu: Pullan 1978; Frantz 1988; Nicholson and Butterworth 1989; Cissé 1995; Vall et al. 2002; Vall and Bayala 2004; for cattle: Croxton and Stollard 1976; Lowman et al. 1976; Buxton 1982; Edmonson et al. 1989; Ferguson et al. 1994; Van der Merve and Stewart 1995; Ezanno 2002; for buffaloes: Ezenwa et al. 2009; Anitha et al. 2010; for camels: Faye et al. 2002; Iglesias et al. 2020; for sheep: Russel et al. 1969; Richard 1997; for goats: Poisot 1988; Honhold et al. 1989; Hervieu et al. 1991; Imadine 1991; Santucci et al. 1991; Ghosh et al. 2019; for horses: Henneke et al. 1983; Carrol and Huntington 1988; Diaw 2013; for donkeys: Pearson and Ouassat 2000; Vall et al. 2001).

Although the importance of BCS as a relevant parameter for assessing the body reserves and welfare of farm animals is widely recognized (for dairy cattle: Edmonson et al. 1989; For zebu: Nicholson and Butterworth 1989; for buffaloes: Carvalho-Delfino et al 2018; for camels: Faye et al 2002; for sheep: Russel et al. 1969; Bocquier et al. 1999; for goats: Battini et al. 2014; for horses: Dugdale et al. 2012), a consistent metric is needed to enhance its precision, accuracy, reproducibility, and repeatability. Thus, the proposed harmonized BCS system presented in this work which standardizes the BCS assessment in a large spectrum of farm animal species reared in tropical and warm regions, address and will reduce the subjectivity of the most used, somehow informal, visual body condition scoring that many authors pointed out (Nicholson and Sayers 1987; Bell et al. 2018).

Although in the standardized BCS system proposed here, the criteria have been harmonized across a wide range of factors, the specificities of the different species in the description of body landmarks have been integrated into the grids. For example, in the case of the buffaloes of Vietnam, the participants of the validation workshop suggested adding a front view (not presented in this paper; see Blanchard et al. 2025a).

Another original aspect of the proposed BCS system is the allocation of scores based on the observation of three anatomical regions of the body from two different perspectives (back and right side). This differs from the majority of proposed BCS systems, which do not consider the neckline (except for Diaw 2013 for horses; and Pearson and Ouassat 2000 for donkeys) and simultaneously the two different perspectives (for zebu: Ayala et al. 1992; for cattle: Van der Merve and Stewart 1995; Frantz 1988; for buffaloes: Anitha et al. 2010; Ezenwa et al. 2009; for camels: Iglesias et al. 2020; for sheep: Richard 1997; for goats: Poisot 1988; Imadine 1991; Ghosh et al. 2019; Honhold et al. 1989; for donkeys: Pearson and Ouassat 2000).

Animal species from tropical regions most often have short-haired coats making their anatomy always clearly visible. Therefore, lumbar palpation is not essential for scoring, as it is for sheep and goats mostly present in temperate zones, which often have woolly or longhaired fleeces (Russel et al. 1969; Hervieu et al. 1991). However, as for small animals, lumbar palpation is an easy observation that can increase the accuracy of the scoring, palpation has been retained in the BCS of sheep and goats (Vall 2025d, e), as has also been suggested by other authors (Poisot 1988; Honhold et al. 1989; Imadine 1991; Richard 1997; Ghosh et al. 2019).

The choice of drawing, instead of pictures (i.e., Pullan 1978; Nicholson and Butterworth 1989; Pearson and Ouassat 2000), allows for a better representation and uniformity in the assessment of the body landmarks at a given level of scoring. This is because it is not easy to get a good picture of an animal in the field, which clearly presents all the criteria assigned to a given score. Therefore, the schematic representation choice (drawing) seems to us to be a benefit of the proposed BCS system. This, while waiting for digital technology to possibly provide solutions that might improve the precision of scoring (e.g., Ferguson et al. 2006; Schroder and Staufenbiel 2006; Halachmi et al. 2008; Bell et al. 2018 for dairy cows; Vieira et al. 2015 for dairy goats).

There is a wide consensus in the subjective nature of the BCS assessment process or method, whatever the animal specie (Nicholson and Sayers 1987; Faye et al. 2002). Therefore, it is important to take the level of caution of each scorer considering the following aspects, which may contribute to increase the accuracy of the assessment: 1) perform regular practical sessions of BCS assessment following the indicated distance from the animal (3 m) for each angle; 2) use of the decisional tree approach, presented in the Fig. 12; 3) take the necessary time to, first of all, respond to the first question (BCS ≥ 3?) through a careful observation of the animal, determining from the start of the assessment in a positive or negative BCS notation.

The standardized BCS system for tropical farm animals presented in this work could be further improved and extended with complementary breeds that are numerous in the tropics, in order to test its robustness and to extend its reach and applicability in a large number of tropical regions and livestock farming conditions.

## Supplementary Information

Below is the link to the electronic supplementary material.

Supplementary file1 (PDF 1965 KB)

Supplementary file2 (PDF 2567 KB)

Supplementary file3 (PDF 1888 KB)

Supplementary file4 (PDF 2756 KB)

Supplementary file5 (PDF 1948 KB)

Supplementary file6 (PDF 2006 KB)

Supplementary file7 (PDF 2497 KB)

Supplementary file8 (PDF 2061 KB)

Supplementary file9 (PDF 2208 KB)

Supplementary file10 (PDF 2166 KB)

## Data Availability

The data and material generated and/or analysed during the current study are available from the corresponding author on a reasonable request.
