# Correction to: Standardized body condition scoring system for tropical farm animals (large ruminants, small ruminants, and equines)

**DOI:** 10.1007/s11250-025-04410-x

**Published:** 2025-04-05

**Authors:** Eric Vall, Mélanie Blanchard, Ollo Sib, Boris Cormary, Eliel González‑García

**Affiliations:** 1 CIRAD, UMR SELMET, 34398 Montpellier, France; 2 SELMET, Univ Montpellier, CIRAD, INRAE, Institut Agro, Montpellier, France; 3 CIRAD, UMR SELMET, Dakar, Senegal; 4 CIRAD, UMR SELMET, Camagüëy, Cuba; 5 INRAE, UMR SELMET, Univ Montpellier, CIRAD, INRAE, Institut Agro, Montpellier, France

**Correction to: Tropical Animal Health and Production**

10.1007/s11250-025-04328-4

The original version of this article contained an error due to a production mistake.

There is a mistake in Fig. 4 of the original article. The drawings in Fig. 4 are not the right ones. Figure 4 reproduces the drawings in Fig. 3 (Body Condition Score grid for Ndama cattle).
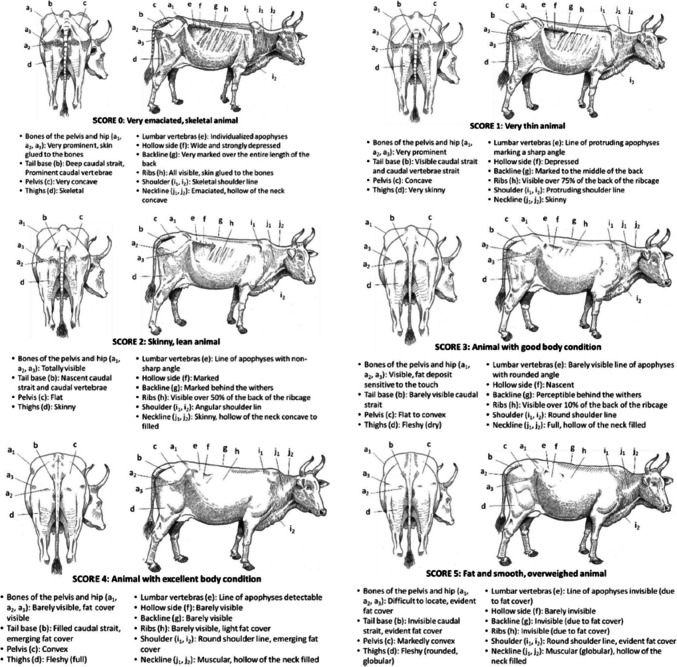


The figure should have appeared as shown below (Body Condition Score grid for crossbred *Bos Taurus* × *Bos indicus* Cuban dairy cattle).
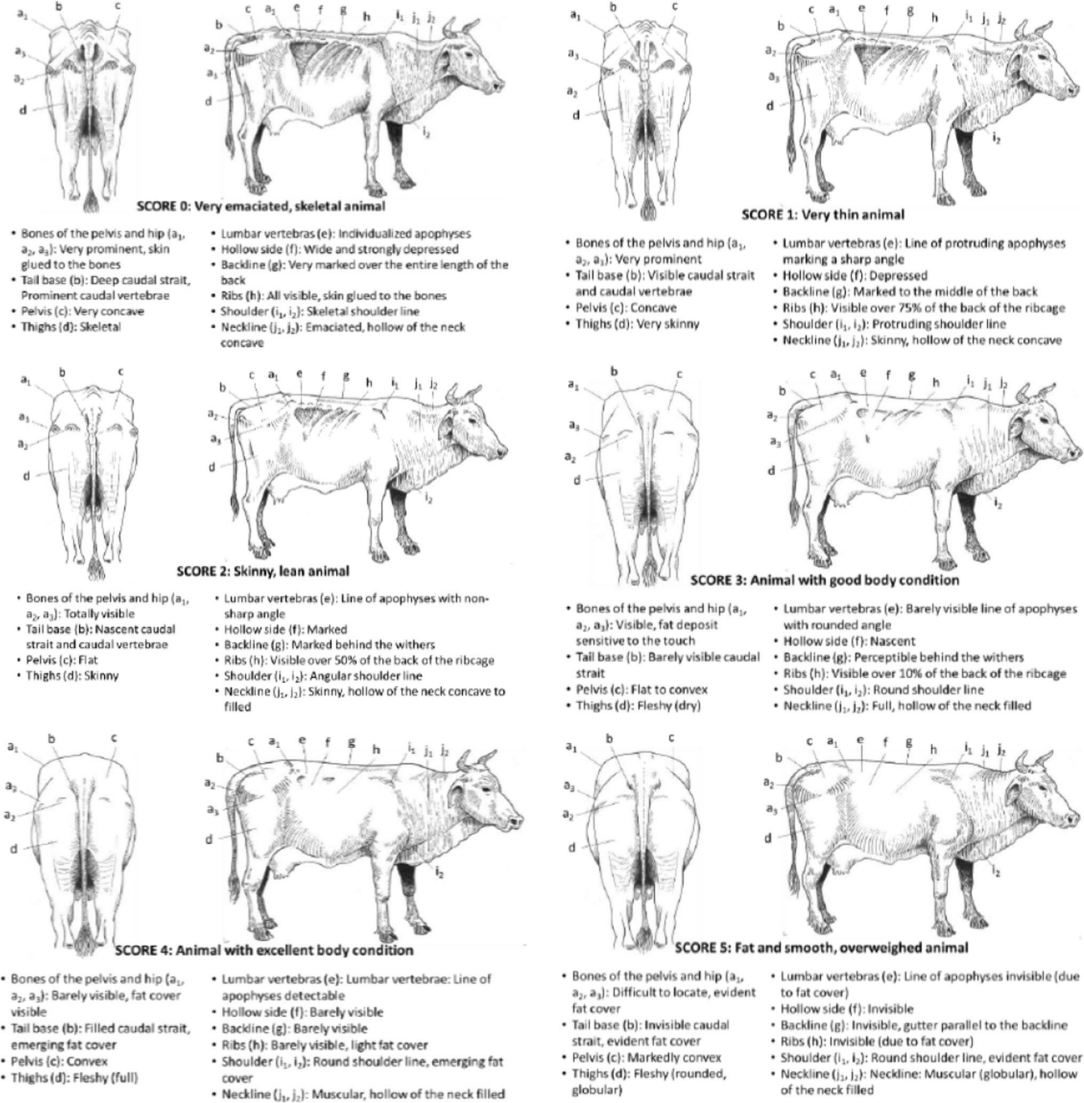


The original article has been corrected.

