# Supplementary material for: Standardized body condition scoring system for tropical farm animals (large ruminants, small ruminants, and equines)
Source: Trop Anim Health Prod. 2025 Mar 7;57(2):106. doi: 10.1007/s11250-025-04328-4 (PMC11889029; doi:10.1007/s11250-025-04328-4)
Supplement: Supplementary file 2 — Supplementary file2 (PDF 2567 KB) [file 11250_2025_4328_MOESM2_ESM.pdf]

Body Condition Scoring Grid for Buffaloes (Vietnam)

Mélanie BLANCHARD[1], Huan LE THI THANH[2], Eric VALL[3]

(1) CIRAD, UMR SELMET, Hanoi, Vietnam
SELMET, Univ Montpellier, CIRAD, INRAE, Institut Agro, Montpellier, France
(2) NIAS, Hanoï, Vietnam
(3) CIRAD, UMR SELMET, F-34398 Montpellier, France
SELMET, Univ Montpellier, CIRAD, INRAE, Institut Agro, Montpellier, France

October 2023

Contents

1 INTRODUCTION 2
2 METHODOLOGY 3
2.1 Principles of construction of the BCS grid 3
2.2 Source of BCS grid drawings 3
3 PRESENTATION OF THE BCS GRID 4
3.1 Anatomical landmarks used to rate the BCS 4
3.2 BCS grid for Buffaloes 6
4 REFERENCES 9
5 ANNEXES 10

1 INTRODUCTION

In tropical countries subject to significant quantitative and qualitative variations in the availability of fodder during the year, the dissatisfaction of feed needs is a frequent situation for animals raired in grazing systems without a sufficient supplementation with feed concentrates.

The Body Condition Scoring (BCS) is a useful way to assess the state of body reserves (subcutaneous fat, muscle mass) which reflects the animal's production (growth, milk, work) and reproduction capacities. The evaluation of the body reserves of an animal through the scoring of their body condition is important to adjust their diet and appreciate their general state of health, as well as their reproductive and production capacity (meat, milk, work, etc.). Managing body reserves is one way of responding to variability in quality and access to feed resources. In females, reserves play an important buffer role during lactation. They can make up for insufficient intakes from the ration. Indeed, the level of production depends on the nutrients provided by the feeds, but also on the animal's body reserves when the feed-based supplies do not fully cover their needs, especially during the dry season. The BCS impacts the interval between two calving. Overly lean cows show a delay in the return of heat after calving, the direct consequence of which is the increase in the parturition interval and consequently a decrease in herd productivity.

The assessment of these reserves through Body Condition Scoring (BCS) represents a management tool for livestock farmers, agricultural advisors and livestock development stakeholders. BCS is a simple, inexpensive and fast method. Several animals can be scored in one session. It allows to compare the BCS of individuals or herds: 1) raised in different production systems or environments, 2) or, during different seasons (dry season and rainy season). BCS can be used as a tool for monitoring and alerting the nutritional level of domestic animal populations. To do this, BCS alert thresholds and a BCS collection and monitoring system must be defined on reasoned samples of animal populations.

It is an easy-to-use field tool. However, a good mastery of the BCS grid as well as a regular practice of scoring are necessary to obtain precise and reproducible ratings. It can also be used as a tool for monitoring and alerting the nutritional level of animal populations. Thus, the farmer can be called at any time to intervene on the feed ration and / or the health of the animal.

The BCS grid currently used in the intervention area of the Beef Cattle 2 project (Figure 1), does not seem to have been developed with reference to the breeds of buffaloes present in Northern Vietnam. This is the reason why we propose this BCS grid adapted to Buffaloes (*Bubalus bubalis*).

In 2020, Vall proposed a standardized BCS scoring system for tropical livestock animals for large animals (cattle, camels), small animals (sheep, and goats) and for donkeys, and horses. This document presents this BCS system applied to the Buffalo.

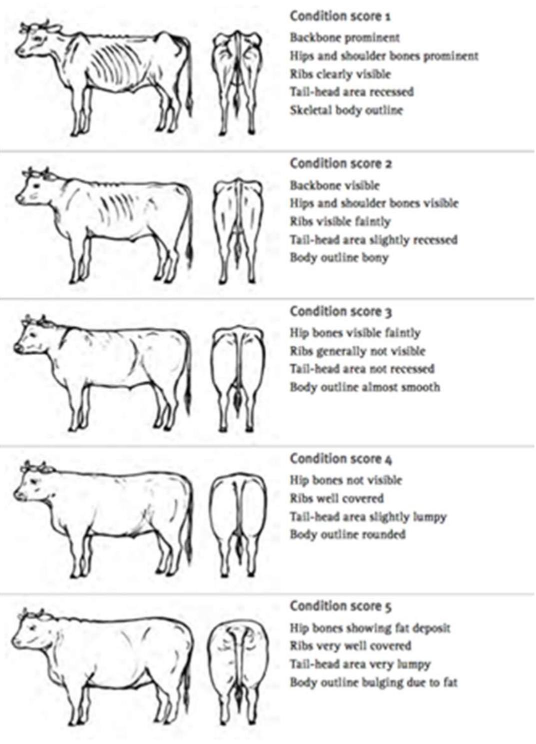

Figure 1: BCS grid currently used in the intervention area of the Beef Cattle 2 project

2 METHODOLOGY

2.1 Principles of construction of the BCS grid

This document aims to present a NEC grid for Buffaloes following the principles and criteria of the harmonized BCS system proposed by Vall (2020) which are as follows:

1) Represent females, because they are the keystone of breeding, because they represent the largest number of breeding animals, and because their body condition is a very good indicator of the good or bad breeding management (in a herd a deterioration of females BCS is indicative of a disorder or problems in rising management);
2) Standardize and simplify the work of assessors by proposing a BCS grid comprising:
   a) A panel of around ten harmonized anatomical points, generally identical for all species, and distributed over three areas of the body: the hindquarters or rump, the thorax and abdomen, the shoulders and the neckline. The detailed description of the anatomical points has been adapted to the case of each species. The ten anatomical landmarks retained are:
      1) Pelvis and hip bone: iliac and ischial spines of the pelvis, and coxo-femoral joint
      2) Base of the tail (caudal strait and caudal vertebrae)
      3) Pelvis
      4) Thighs
      5) Lumbar vertebrae (transverse processes of the lumbar vertebrae)
      6) Hollow side
      7) Backline (spinous processes of the dorsal vertebrae)
      8) Ribs
      9) Shoulder: shoulder blade, and humerus joint
      10) Neckline (hollow of the neck and general appearance)
   b) The same range of scoring (from 0 to 5) for all the species. Beyond 6 levels, the distinction between two BCS levels becomes very subjective and below that it lacks of acuity. The 6-level rating systems are the most widespread in the literature;
   c) A "back BCS" and a "right-side BCS", with a Final BCS corresponding to the average of the two;
   d) During a workshop validation of the BCS grid the actors of the buffalo value chain of Vietnam proposed to add a "front view BCS" for Buffaloes to fine tune the final BCS score;

2.2 Source of BCS grid drawings

As it is not easy to have animals in the same position, we chose to create a BCS grid with drawings representing a typical animal in the different BCS levels and from three perspectives angles (side view, back view, plus front view). The grid was developed from a series of photos of Buffaloes; part of which is presented in the Annex. The drawings were made in Indian ink by Eric Vall.

Thanks to Mr Lê Tiến Dũng from NIAS and Ms Dong Thi Na du CIRAD (flagship facilitator in Dien Bien) who provided us with a series of photos taken on 11 Buffaloes in the Dien Bien area. The animals were photographed from three perspectives (side view, back view, plus front view) at approximately 3 m of distance.

3 PRESENTATION OF THE BCS GRID

3.1 Anatomical landmarks used to rate the BCS

The scoring is made by visual observations of the BCS landmarks distributed on the three parts of the body, represented on the Figure 2:

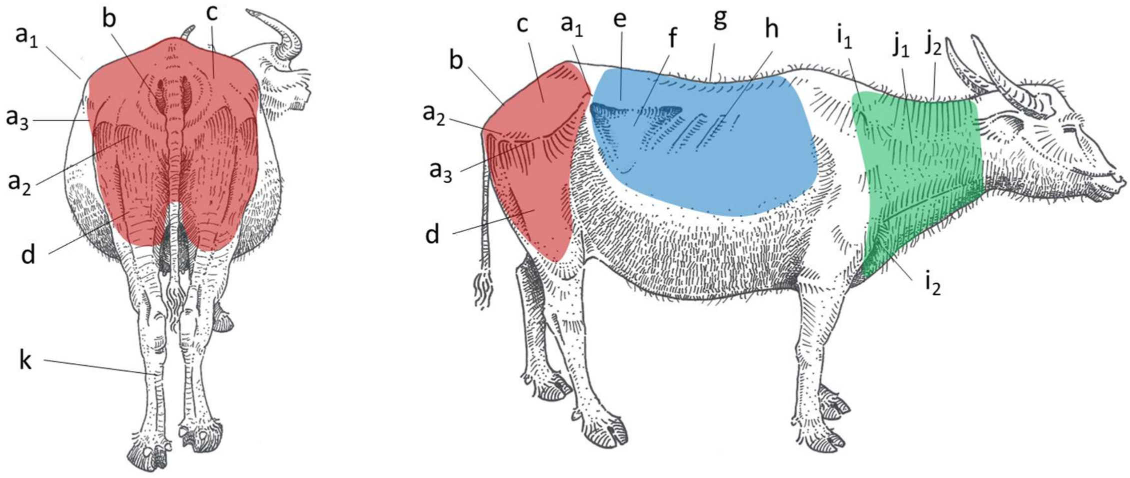

Hindquarters (rump): Bones of the pelvis and hip: iliac (a_1) and ischial (a_2) spines of the pelvis, coxo-femoral joint (a_3); **tail base:** tail base and caudal vertebrae (b); **Pelvis:** pelvis cover (c); **Thighs:** thigh covers (d); foot tendon aspect (k)

Thorax and abdomen: Lumbar vertebrae: transverse processes of lumbar vertebrae (e); **Hollow side:** hollow of the side (f); **Backline:** spinous processes of the dorsal vertebrae (g), **Ribs:** ribs appearance (h)

Shoulder and neck: Shoulder: shoulder blade (i_1), humerus joint (i_2); **Neckline:** hollow of the neck (j_1), general appearance (j_2)

Figure 2. BCS landmarks for Buffaloes (back and right-side views)

The attendees to the workshop validation of the BCS grid proposed to add a land mark on tendon feet appearance (see landmark K).

The attendees to the workshop validation of the BCS grid proposed to add a front view scoring for buffaloes (chest scoring) to fine tune the scoring (Figure 3), plus precisions on the appearance of the feet tendons; and precisions on the aspect of the shoulders and the chest in side view;

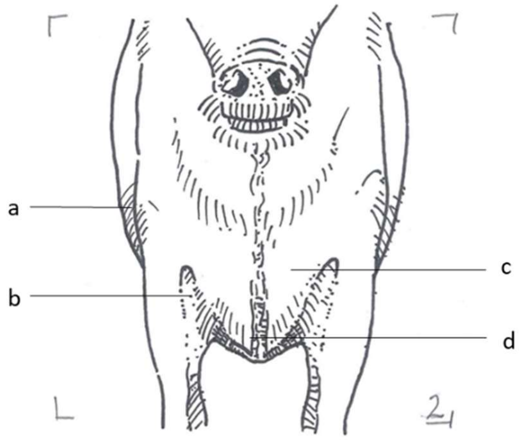

a: shoulders
b: hollow between the chest and the forelimbs
c: concavity or convexity of the chest
d: tip of the chest (sternum)

Figure 3. BCS landmarks for Buffaloes (front view for a chest score)

3.2 BCS grid for Buffaloes

Table 1. BCS grid for Buffaloes

	Hindquarters (rump)				Thorax and abdomen				Shoulders and neck		Front view
**Score**	**Bones of the pelvis and hip**	**Tail base**	**Pelvis**	**Thighs Feet tendons**	**Lumbar vertebrae**	**Hollow side**	**Backline**	**Ribs**	**Shoulder**	**Neckline**	**Appearance of the chest**
**0**	Very prominent, skin glued to the bones	Deep caudal strait, Prominent caudal vertebrae	Very concave	Skeletal Very thin	Individualized apophyses	Wide and strongly depressed	Very marked over the entire length of the back	All visible	Skeletal shoulder line	Emaciated, hollow of the neck concave	Skeletal and concave
**1**	Very prominent	Visible caudal strait and caudal vertebrae	Concave	Very skinny Very skinny	Line of protruding apophyses marking a sharp angle	Depressed	Marked to the middle of the back	Visible over 50% of the back of the ribcage	Protruding shoulder line	Skinny, hollow of the neck concave	Concave
**2**	Totally visible	Nascent caudal strait and caudal vertebrae	Flat	Skinny; Thin	Line of apophyses with non-sharp angle	Marked	Marked behind the withers	Visible over 25% of the back of the ribcage	Angular shoulder line	Skinny, hollow of the neck concave to filled	Slightly concave
**3**	Visible, fat deposit sensitive to the touch	Barely visible caudal strait	Flat to convex	Fleshy (dry) Thick	Barely visible line of apophyses with rounded angle	Nascent	Perceptible behind the withers	Visible over 10% of the back of the ribcage	Round shoulder line	Full, hollow of the neck filled	Slightly convex
**4**	Barely visible, fat cover visible	Filled caudal strait, emerging fat cover	Convex	Fleshy (full) Thick	Line of apophyses detectable	Barely visible	Barely visible	Invisible, light fat cover	Round shoulder line, emerging fat cover	Muscular, hollow of the neck filled	Convex
**5**	Difficult to locate, evident fat cover	Invisible caudal strait, evident fat cover	Markedly convex	Fleshy (rounded); Very thick	Line of apophyses invisible (due to fat cover)	Invisible	Invisible (due to fat cover)	Invisible (due to evident fat cover)	Round shoulder line, evident fat cover	Muscular (globular), hollow of the neck filled	Very convex

Figure 4. BCS grid for buffaloes (commented drawings): scoring from 0 to 2

Score	Back view	Right side view
**Score 0: Skeletal animal**<br><br>**Bones of the pelvis and hip:** Very prominent, skin glued to the bones<br>**Tail base:** Deep caudal strait, Prominent caudal vertebrae<br>**Pelvis:** Very concave<br>**Thighs; & Feet tendons:** Skeletal; Very thin<br>**Lumbar vertebrae:** Individualized apophyses<br>**Hollow side:** Wide and strongly depressed<br>**Backline:** Very marked over the entire length of the back<br>**Ribs:** All visible<br>**Shoulder:** Skeletal shoulder line<br>**Neckline:** Emaciated, hollow of the neck concave	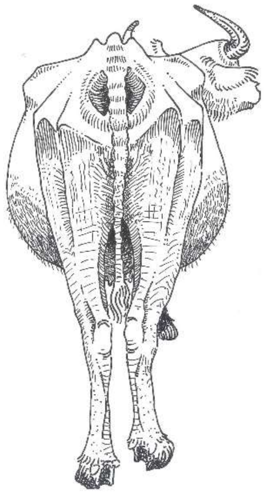	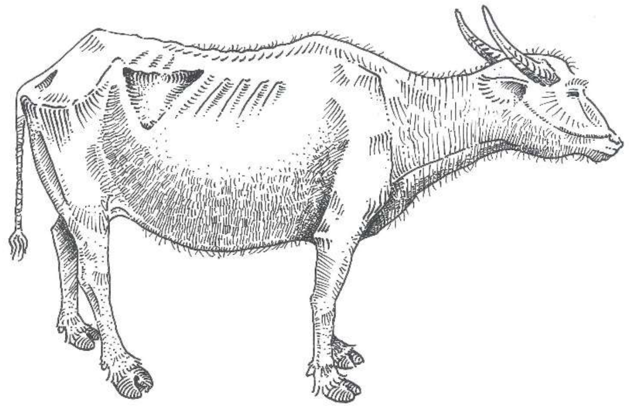
**Score 1: Very thin animal**<br><br>**Bones of the pelvis and hip:** Very prominent<br>**Tail base:** Visible caudal strait and caudal vertebrae<br>**Pelvis:** Concave<br>**Thighs; & Feet tendons:** Very skinny; Very skinny<br>**Lumbar vertebrae:** Line of protruding apophyses marking a sharp angle<br>**Hollow side:** Depressed<br>**Backline:** Marked to the middle of the back<br>**Ribs:** Visible over 50% of the back of the ribcage<br>**Shoulder:** Protruding shoulder line<br>**Neckline:** Skinny, hollow of the neck concave	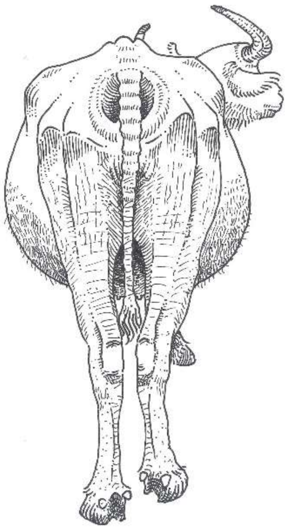	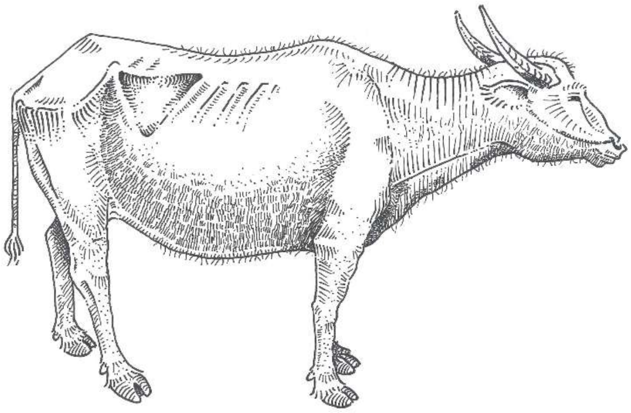
**Score 2: Skinny, lean animal**<br><br>**Bones of the pelvis and hip:** Totally visible<br>**Tail base:** Nascent caudal strait and caudal vertebrae<br>**Pelvis:** Flat<br>**Thighs; & Feet tendons:** Skinny; Thin<br>**Lumbar vertebrae:** Line of apophyses with non-sharp angle<br>**Hollow side:** Marked<br>**Backline:** Marked behind the withers<br>**Ribs:** Visible over 25% of the back of the ribcage<br>**Shoulder:** Angular shoulder line<br>**Neckline**: Skinny, hollow of the neck concave to filled	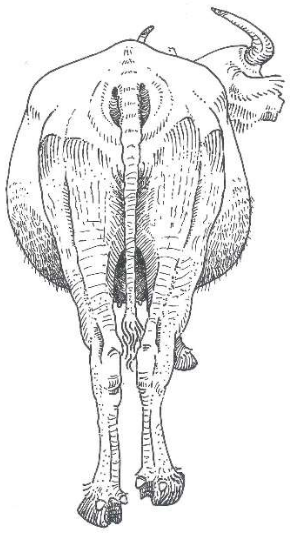	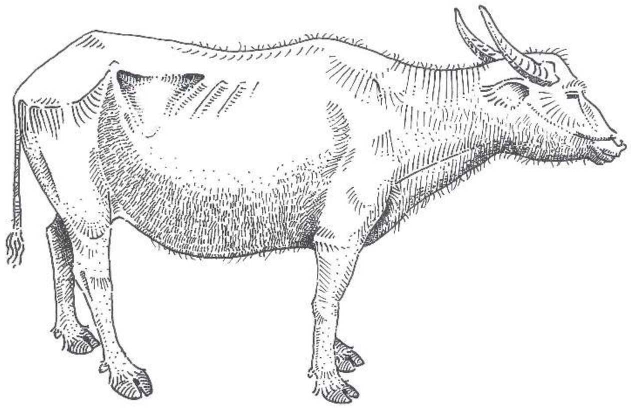

Figure 5. BCS grid for buffaloes (commented drawings): scoring from 3 to 5

Score	Back view	Right side view
**Score 3: Animal with good body condition**<br><br>**Bones of the pelvis and hip:** Visible, fat deposit sensitive to the touch<br>**Tail base:** Barely visible caudal strait<br>**Pelvis:** Flat to convex<br>**Thighs; & Feet tendons:** Fleshy (dry); Thick<br>**Lumbar vertebrae:** Barely visible line of apophyses with rounded angle<br>**Hollow side:** Nascent<br>**Backline:** Perceptible behind the withers<br>**Ribs:** Visible over 10% of the back of the ribcage<br>**Shoulder:** Round shoulder line<br>**Neckline:** Full, hollow of the neck filled	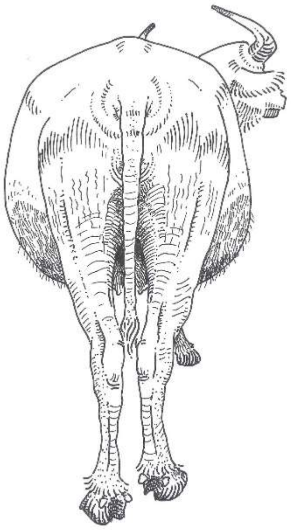	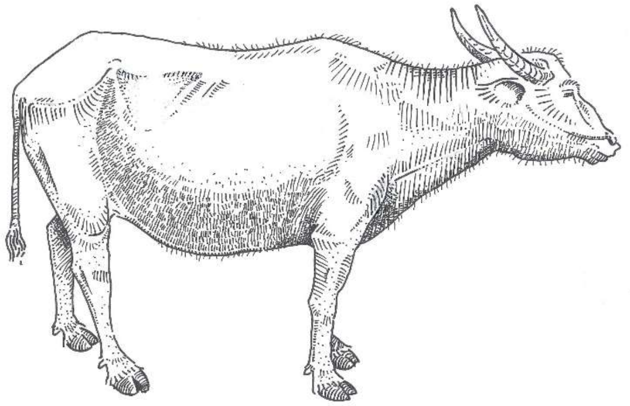
**Score 4: Animal with excellent body condition**<br><br>**Bones of the pelvis and hip:** Barely visible, fat cover visible<br>**Tail base:** Filled caudal strait, emerging fat cover<br>**Pelvis:** Convex<br>**Thighs; & Feet tendons**: Fleshy (full); Thick<br>**Lumbar vertebrae:** Line of apophyses detectable<br>**Hollow side:** Barely visible<br>**Backline:** Barely visible<br>**Ribs:** Invisible, light fat cover<br>**Shoulder:** Round shoulder line, emerging fat cover<br>**Neckline:** Muscular, hollow of the neck filled	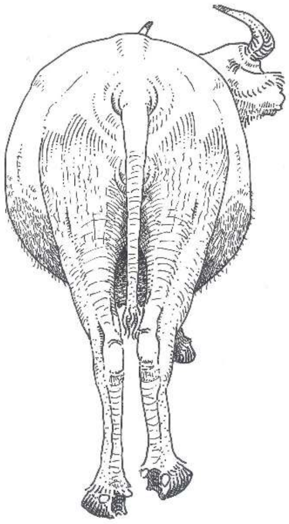	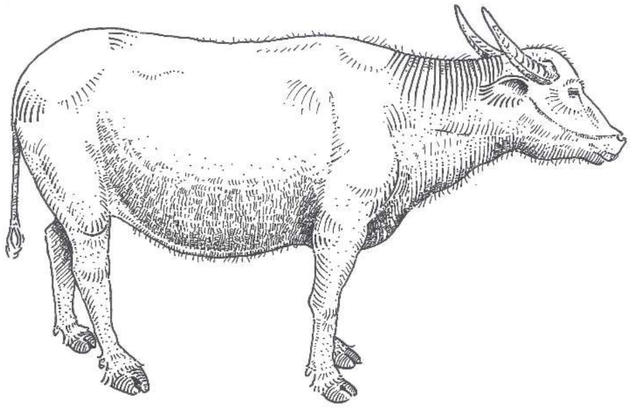
**Score 5: Fat and smooth, overweight animal**<br><br>**Bones of the pelvis and hip:** Difficult to locate, evident fat cover<br>**Tail base:** Invisible caudal strait, evident fat cover<br>**Pelvis:** Markedly convex<br>**Thighs & Feet tendons:** Fleshy (rounded); Very thick<br>**Lumbar vertebrae**: Line of apophyses invisible (due to fat cover)<br>**Hollow side:** Invisible<br>**Backline:** Invisible (due to fat cover)<br>**Ribs:** Invisible (due to evident fat cover)<br>**Shoulder:** Round shoulder line, evident fat cover<br>**Neckline:** Muscular (globular), hollow of the neck filled	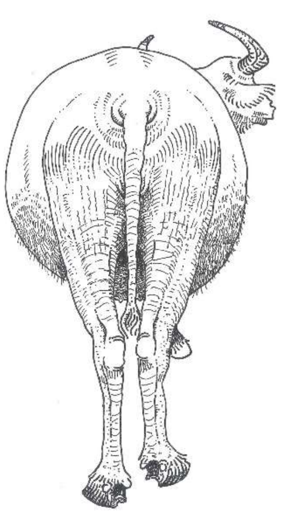	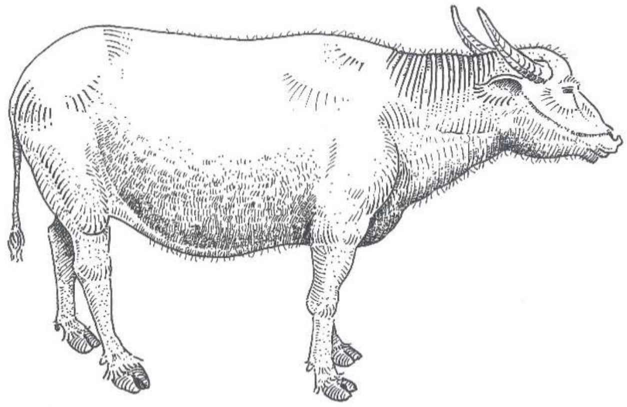

Figure 6. BCS grid for buffaloes: chest scoring from a front view (scoring from 0 to 5)

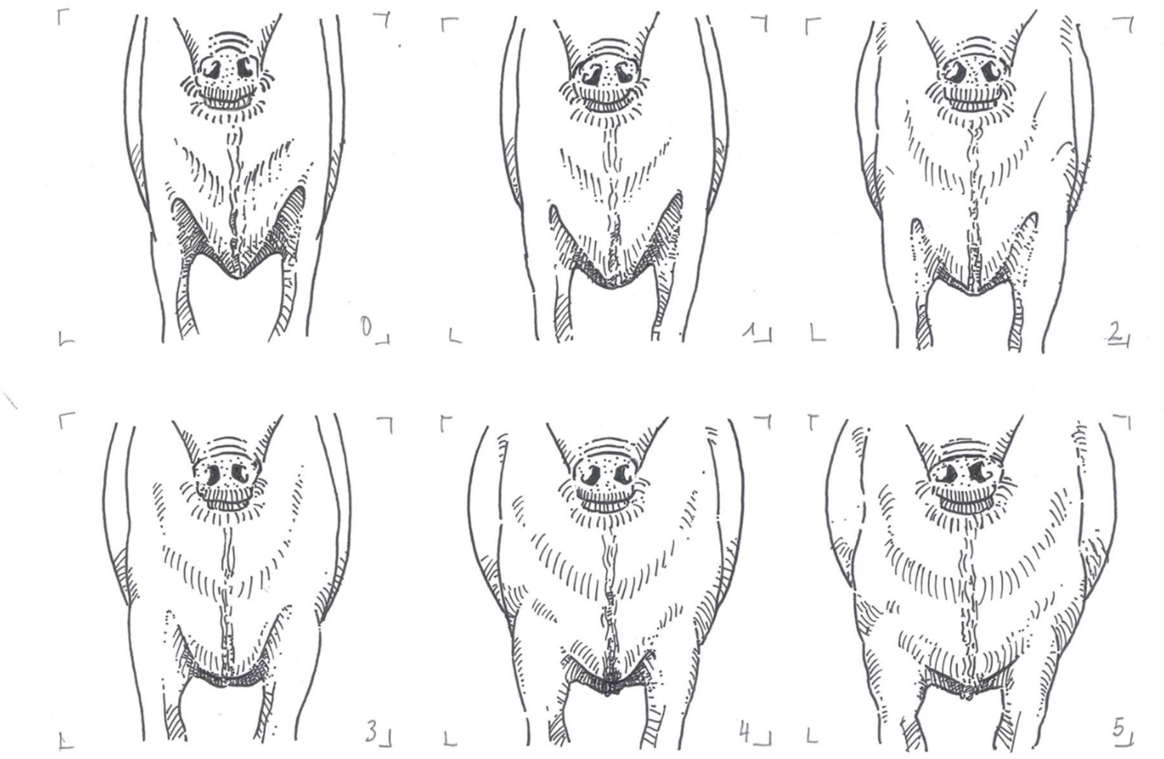

However, it must be admitted that the face scoring is not easy to do because it is difficult to hold an animal facing you without its owner holding it by the muzzle. Moreover, we can clearly see in the photos of the Annexe 3 that buffaloes are rarely in the right position.

4 REFERENCES

Vall E., 2020. Guide harmonisé de Notation de l'Etat Corporel (NEC) pour les animaux de ferme du Sahel : Ruminants de grande taille (Bovins, Camelins) et de petite taille (Ovins, Caprins) et équidés (Asins et Equins). CILSS, Cirad, Montpellier, France, 43 p.

5 ANNEXES

Annexe 1. Pictures of Buffaloes with BCS varying from 0 to 2

Score	Back view	Right side view
**Score 0: Skeletal animal**	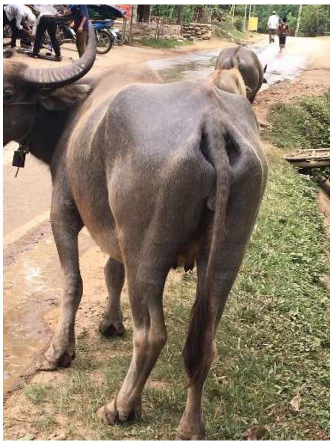	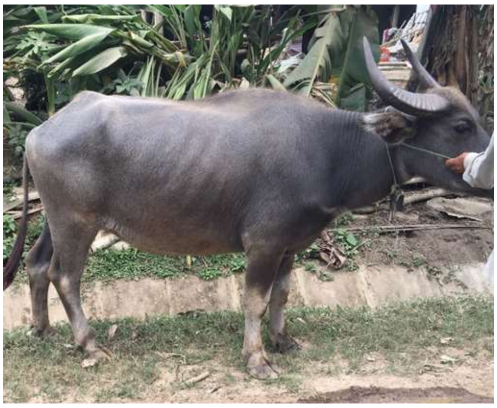
**Score 1: Very thin animal**	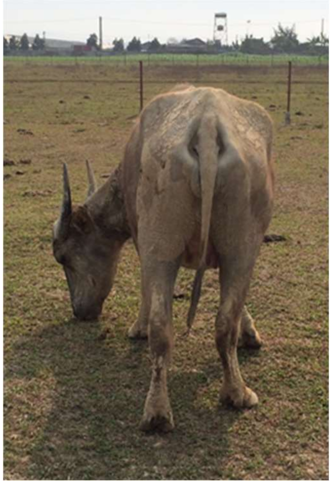	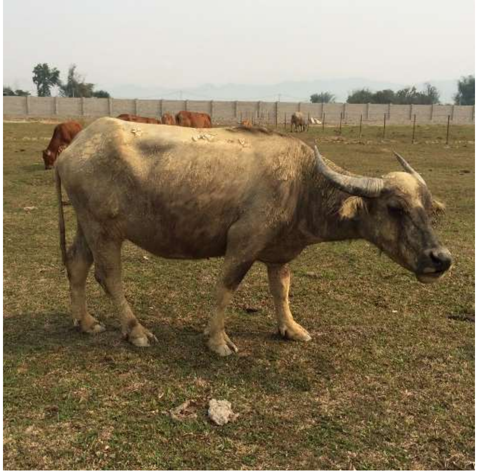
**Score 2: Skinny, lean animal**	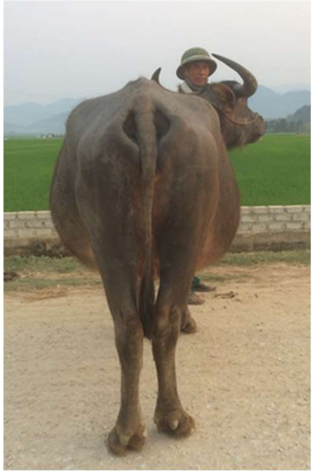	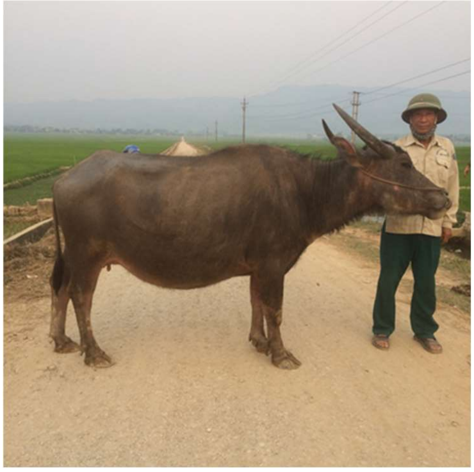

Annexe 2. Pictures of Buffaloes with BCS varying from 3 to 5

Score	Back view	Right side view
**Score 3: Animal with good body condition**	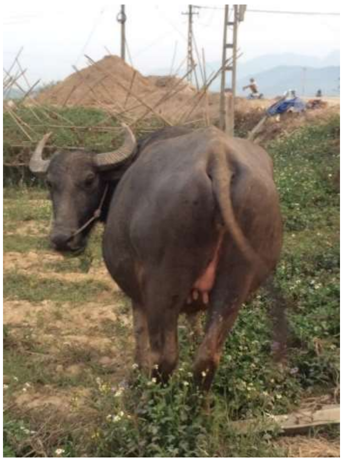	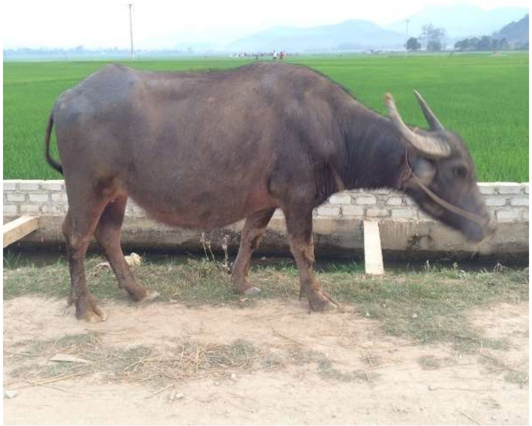
**Score 4: Animal with excellent body condition**	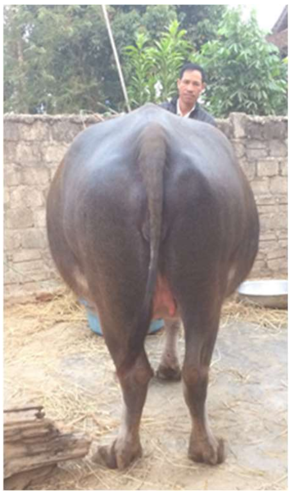	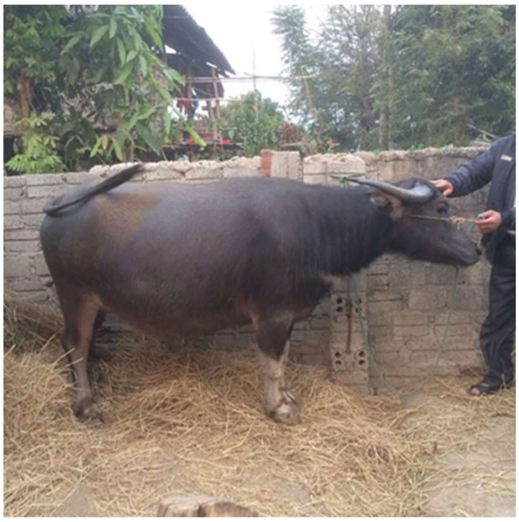
**Score 5: Fat and smooth animal overweight animal**	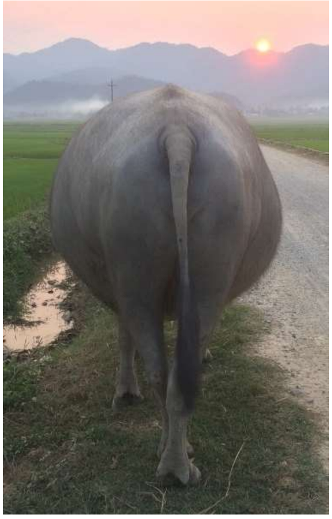	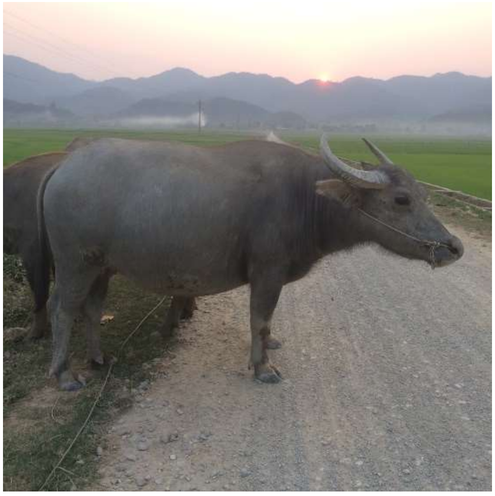

Annexe 3. Front view of Buffaloes with BCS varying from 0 to 3

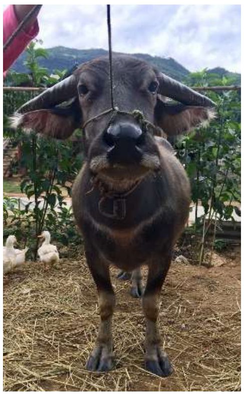<br>0	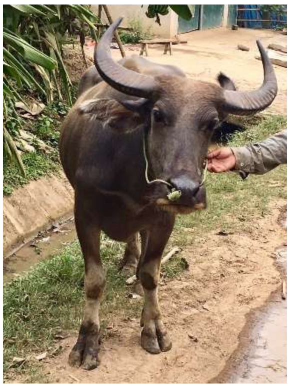<br>1	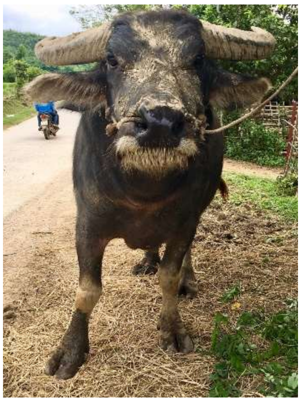<br>2
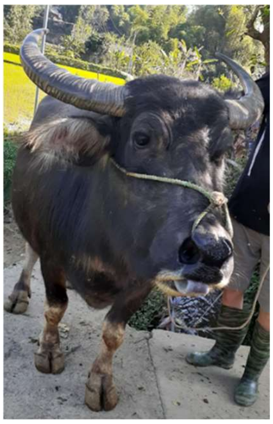<br>3	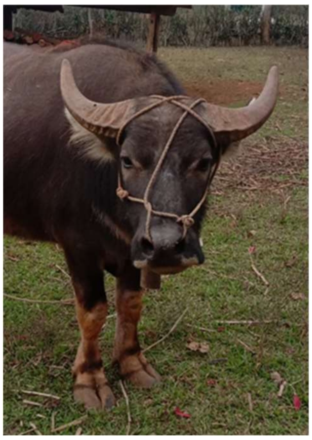<br>4	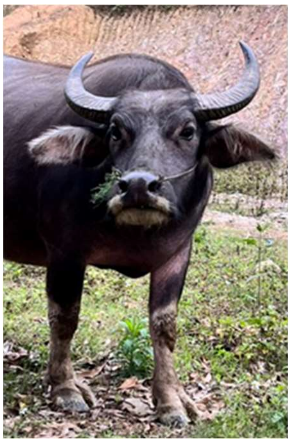<br>5
