# Supplementary material for: Standardized body condition scoring system for tropical farm animals (large ruminants, small ruminants, and equines)
Source: Trop Anim Health Prod. 2025 Mar 7;57(2):106. doi: 10.1007/s11250-025-04328-4 (PMC11889029; doi:10.1007/s11250-025-04328-4)
Supplement: Supplementary file 6 — Supplementary file6 (PDF 2006 KB) [file 11250_2025_4328_MOESM6_ESM.pdf]

Body Condition Scoring Grid for Goat (Sahel and Savannah areas)

Eric VALL[1]

(1) CIRAD, UMR SELMET, F-34398 Montpellier, France.
SELMET, Univ Montpellier, CIRAD, INRAE, Institut Agro, Montpellier, France.

October 2023

Contents

1 INTRODUCTION 2

2 METHODOLOGY 3

2.1 Principles of construction of the BCS grid 3

2.2 Source of BCS grid drawings 3

3 PRESENTATION OF THE BCS GRID 4

3.1 Anatomical landmarks used to rate the BCS 4

3.2 BCS grid for goats 5

4 REFERENCES 8

5 ANNEXES 9

1 INTRODUCTION

In tropical countries subject to significant quantitative and qualitative variations in the availability of fodder during the year, the dissatisfaction of feed needs is a frequent situation for animals maintained on rangelands without a sufficient supplementation with feed concentrates.

The Body Condition Scoring (BCS) is a useful way to assess the state of body reserves (subcutaneous fat, muscle mass) which reflects the animal's production (growth, milk, work) and reproduction capacities. The evaluation of the body reserves of an animal through the scoring of their body condition is important to adjust their diet and appreciate their general state of health, as well as their reproductive and production capacity (meat, milk, work, etc.). Managing body reserves is one way of responding to variability in quality and access to feed resources. In females, reserves play an important buffer role during lactation. They can make up for insufficient intakes from the ration. Indeed, the level of production depends on the nutrients provided by the feeds, but also on the animal's body reserves when the feed-based supplies do not fully cover their needs, especially during the dry season. The BCS impacts the interval between births. Overly lean females show a delay in the return of heat after giving birth, the direct consequence of which is the increase in the parturition interval and consequently a decrease in herd productivity.

The assessment of these reserves through Body Condition Scoring (BCS) represents a management tool for livestock farmers, agricultural advisors and livestock development stakeholders. BCS is a simple, inexpensive and fast method. Several animals can be scored in one session. It allows to compare the BCS of individuals or herds: 1) raised in different production systems or environments, 2) or, during different seasons (dry season and rainy season). BCS can be used as a tool for monitoring and alerting the nutritional level of domestic animal populations. To do this, BCS alert thresholds and a BCS collection and monitoring system must be defined on reasoned samples of animal populations.

It is an easy-to-use field tool. However, a good mastery of the BCS grid as well as a regular practice of scoring are necessary to obtain precise and reproducible ratings. It can also be used as a tool for monitoring and alerting the nutritional level of animal populations. Thus, the farmer can be called at any time to intervene on the feed ration and / or the health of the animal.

Few studies have been carried out for developing a BCS grid, specially devoted and applicable to Sub-Saharan goats in Africa. Existing BCS systems for goats are based on a four-points BCS grid (Imadine, 1991), which has been adapted from systems previously developed for temperate goats (Morand-Fehr and Branca, 1987; Hervieu et al., 1991). Such a system includes examining the lumbar region (transverse and spinous apophyses, fat thickness, and skin appearance), the sternal region (fat thickness) and the general appearance of the animal (ribs, bones protrusions in the rump, transverse and spinous apophyses; Imadine, 1991; Cisse 1995; Cisse et al., 1992, 1994). The criteria retained by those authors includes observation from the rear and from the flank of the animal.

In 2020, Vall proposed a standardized BCS scoring system for tropical livestock animals for large animals (cattle, camels), small animals (sheep, and goats) and for donkeys, and horses. This document presents this BCS system applied to the Sahelian goat.

2 METHODOLOGY

2.1 Principles of construction of the BCS grid

This document aims to present a BCS grid for goats following the principles and criteria of the harmonized BCS system proposed by Vall (2020) which are as follows:

1) Represent females, because they are the keystone of breeding, because they represent the largest number of breeding animals, and because their body condition is a very good indicator of the good or bad breeding management (in a herd a deterioration of females BCS is indicative of a disorder or problems in rising management);
2) Standardize and simplify the work of assessors by proposing a BCS grid comprising:
    a) A panel of around ten harmonized anatomical points, generally identical for all species, and distributed over three areas of the body: the hindquarters or rump, the thorax and abdomen, the shoulders and the neckline. The detailed description of the anatomical points has been adapted to the case of each species. The ten anatomical landmarks retained are:
        1) Pelvis and hip bone: iliac and ischial spines of the pelvis, and coxo-femoral joint
        2) Base of the tail (caudal strait and caudal vertebrae)
        3) Pelvis
        4) Thighs
        5) Lumbar vertebrae (transverse processes of the lumbar vertebrae)
        6) Hollow side
        7) Backline (spinous processes of the dorsal vertebrae)
        8) Ribs
        9) Shoulder: shoulder blade, and humerus joint
        10) Neckline (hollow of the neck and general appearance)
    b) The same range of scoring (from 0 to 5) for all the species. Beyond 6 levels, the distinction between two BCS levels becomes very subjective and below that it lacks of acuity. The 6-level rating systems are the most widespread in the literature;
    c) A “back BCS” and a “right-side BCS”, with a Final BCS corresponding to the average of the two;

2.2 Source of BCS grid drawings

As it is not easy to have animals in the same position, we chose to create a BCS grid with drawings representing a typical animal in the different BCS levels and from two perspectives angles (side view, back view). The grid was developed from a series of photos of goats; part of which is presented in the Annex. The drawings were made in Indian ink by Eric Vall.

The photos were taken by Eric Vall in Burkina Faso and Senegal. The animals were photographed from two perspectives (side view, back view) at approximately 3 m of distance.

3 PRESENTATION OF THE BCS GRID

3.1 Anatomical landmarks used to rate the BCS

The scoring is made by visual observations of the BCS landmarks distributed on the three parts of the body, represented on the Figure 2:

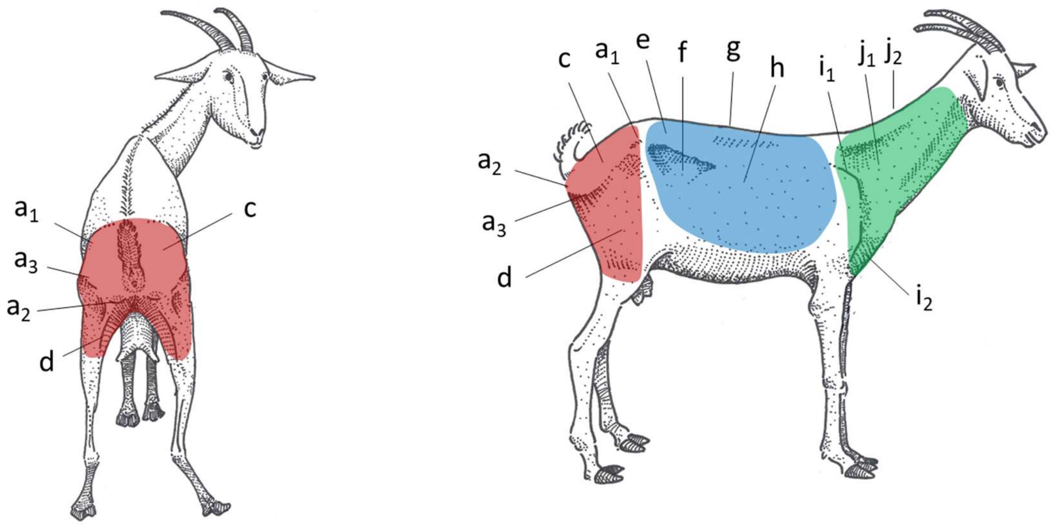

Hindquarters (rump): Bones of the pelvis and hip: iliac (a_1) and ischial (a_2) spines of the pelvis, coxo-femoral joint (a3); **tail base:** tail base and caudal vertebrae (b); **Pelvis:** pelvis cover (c); **Thighs:** thigh covers (d)

Thorax and abdomen: Lumbar vertebrae: transverse processes of lumbar vertebrae (e); **Hollow side:** hollow of the side (f); **Backline:** spinous processes of the dorsal vertebrae (g), **Ribs:** ribs appearance (h)

Shoulder and neck: Shoulder: shoulder blade (i_1), humerus joint (i_2); **Neckline:** hollow of the neck (j_1), general appearance (j_2)

Figure 1. BCS landmarks for a goat (back and right-side views)

In the current proposed BCS Grid, the aspect (assessment) of the tail base was not taken into account since we do not observe a crossing of the uro-genital depression when the body condition of the goat deteriorates. To confirm the flank score, we suggest performing a lumbar palpation to complete the scoring.

3.2 BCS grid for goats

Table 1. BCS grid for goats

	**Hindquarters (rump)**				**Thorax and abdomen**					**Shoulder and neck**	
**Score**	**Bones of the pelvis and hip**	**Tail base**	**Pelvis**	**Thighs**	**Lumbar palpation**	**Lumbar vertebrae**	**Hollow side**	**Backline**	**Ribs**	**Shoulder**	**Neckline**
**0**	Very prominent, skin glued to the bones	Prominent caudal vertebrae	Very concave	Skeletal	Fingers fit easily between the transverse processes. No thickness of fat. Dry skin does not roll off the hand	Individualized apophyses	Wide and strongly depressed	Very marked over the entire length of the back	Visible over 50% of the back of the ribcage	Skeletal shoulder line	Emaciated, hollow of the neck concave
**1**	Very prominent	Visible caudal vertebrae	Concave	Very skinny	Fingers fit easily between the transverse apophyses. Without fat thickness of fat. Dry skin does not roll off the hand	Line of protruding apophyses marking a sharp angle	Depressed	Marked on the front of the back	Visible over 25% of the back of the ribcage	Protruding shoulder line	Neckline: Skinny, hollow of the neck concave
**2**	Totally visible	Nascent caudal vertebrae	Flat	Skinny	Fingers insert between transverse apophyses. Light fat thickness. Skin does not roll out of hand.	Line of apophyses with non-sharp angle	Marked	Slightly visible behind the withers	Invisible	Angular shoulder line	Skinny, hollow of the neck concave to filled
**3**	Visible, fat deposit sensitive to the touch	Barely visible caudal vertebrae	Flat to convex	Fleshy (dry)	Fingers insert between transverse apophyses with slight pressure. Average fat thickness. Skin rolls lightly under hand.	Barely visible line of apophyses with rounded angle	Nascent	Perceptible behind the withers	Invisible, nascent fat cover	Round shoulder line	Full, hollow of the neck filled
**4**	Barely visible, fat cover visible	Emerging fat cover	Convex	Fleshy (full)	Fingers insert between the transverse apophyses with pressure. Medium to strong fat thickness. Skin rolls under hand	Line of apophyses detectable	Barely visible	Barely visible	Invisible, light fat cover	Shoulder: Round shoulder line, emerging fat cover	Neckline: Muscular, hollow of the neck filled
**5**	Difficult to locate, evident fat cover	Evident fat cover	Markedly convex	Fleshy (rounded, globular)	Fingers insert between transverse apophyses with strong pressure. Strong fat thickness. Skin rolls under hand	Line of apophyses invisible (due to fat cover)	Invisible	Invisible (due to fat cover)	Invisible (due to evident fat cover)	Round shoulder line, evident fat cover	Muscular (globular), hollow of the neck filled

Figure 2. BCS grid for goats (commented drawings): scoring from 0 to 2

Score	Back view	Right side view
**SCORE 0: Very emaciated, skeletal animal**<br><br>**Bones of the pelvis and hip:** Very prominent, skin glued to the bones<br>**Tail base:** Prominent caudal vertebrae<br>**Pelvis:** Very concave<br>**Thighs:** Skeletal<br>**Lumbar vertebrae:** Individualized apophyses<br>**Hollow side:** Wide and strongly depressed<br>**Backline:** Very marked over the entire length of the back<br>**Ribs:** Visible over 50% of the back of the ribcage<br>**Shoulder:** Skeletal shoulder line<br>**Neckline:** Emaciated, hollow of the neck concave	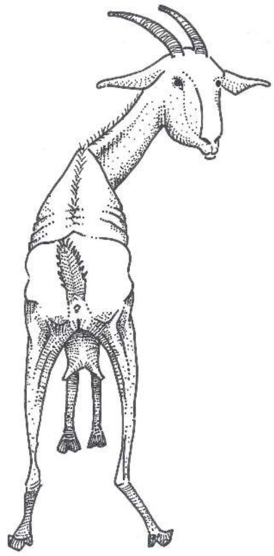	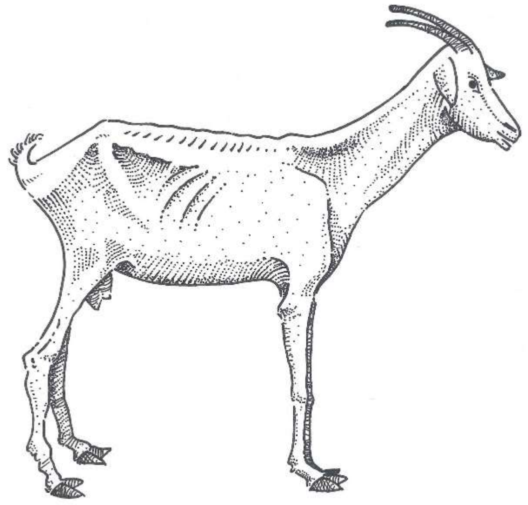
**SCORE 1: Very thin animal**<br><br>**Bones of the pelvis and hip:** Very prominent<br>**Tail base:** Visible caudal vertebrae<br>**Pelvis:** Concave<br>**Thighs:** Very skinny<br>**Lumbar vertebrae:** Line of protruding apophyses marking a sharp angle<br>**Hollow side:** Depressed<br>**Backline:** Marked on the front of the back<br>**Ribs:** Visible over 25% of the back of the ribcage<br>**Shoulder:** Protruding shoulder line<br>**Neckline:** Skinny, hollow of the neck concave	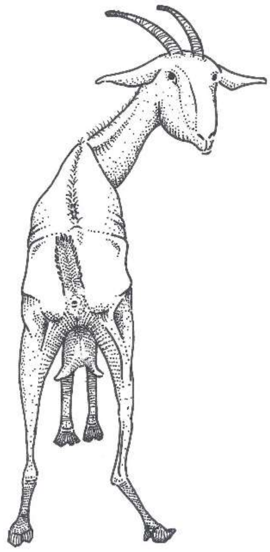	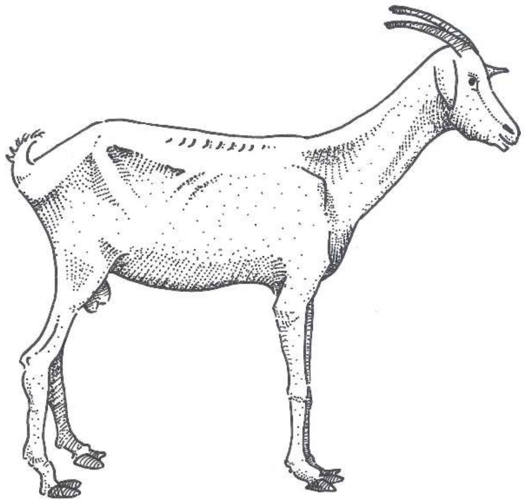
**SCORE 2: Skinny, lean animal**<br><br>**Bones of the pelvis and hip:** Totally visible<br>**Tail base:** Nascent caudal vertebrae<br>**Pelvis:** Flat<br>**Thighs:** Skinny<br>**Lumbar vertebrae:** Line of apophyses with non-sharp angle<br>**Hollow side:** Marked<br>**Backline:** Slightly visible behind the withers<br>**Ribs:** Invisible<br>**Shoulder:** Angular shoulder line<br>**Neckline:** Skinny, hollow of the neck concave to filled	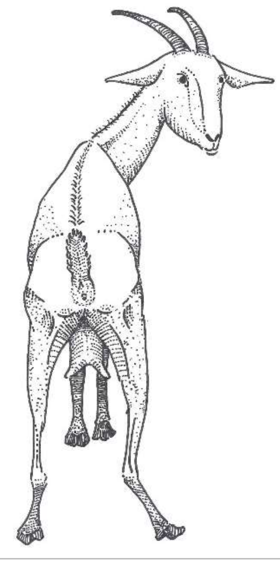	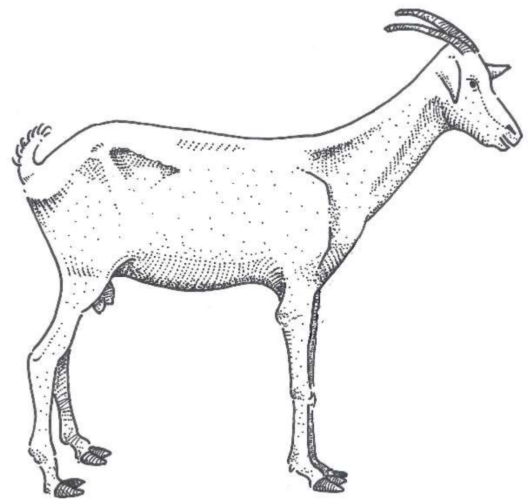

Figure 3. BCS grid for goats (commented drawings): scoring from 3 to 5

Score	Back view	Right side view
**SCORE 3: Animal with good body condition**<br><br>**Bones of the pelvis and hip**: Visible, fat deposit sensitive to the touch<br>**Tail base:** Barely visible caudal vertebrae<br>**Pelvis:** Flat to convex<br>**Thighs:** Fleshy (dry)<br>**Lumbar vertebrae:** Barely visible line of apophyses with rounded angle<br>**Hollow side:** Nascent<br>**Backline:** Perceptible behind the withers<br>**Ribs:** Invisible, nascent fat cover<br>**Shoulder:** Round shoulder line<br>**Neckline:** Full, hollow of the neck filled	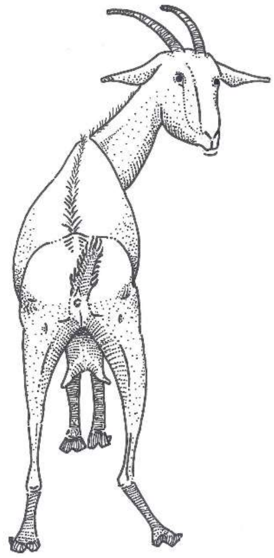	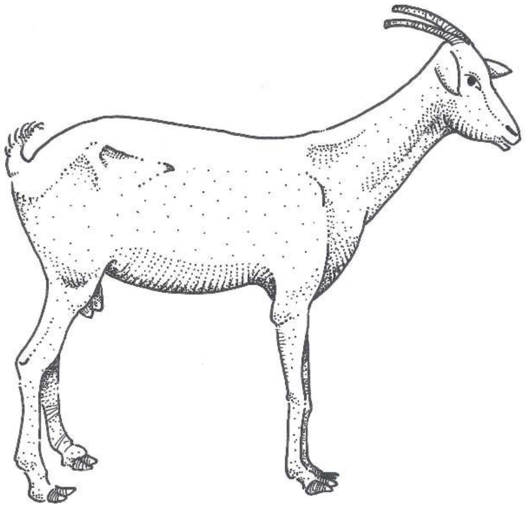
**SCORE 4: Animal with excellent body condition**<br><br>**Bones of the pelvis and hip:** Barely visible, fat cover visible<br>**Tail base:** Emerging fat cover<br>**Pelvis:** Convex<br>**Thighs:** Fleshy (full)<br>**Lumbar vertebrae:** Line of apophyses detectable<br>**Hollow side:** Barely visible<br>**Backline:** Barely visible<br>**Ribs:** Invisible, light fat cover<br>**Shoulder:** Round shoulder line, emerging fat cover<br>**Neckline:** Muscular, hollow of the neck filled	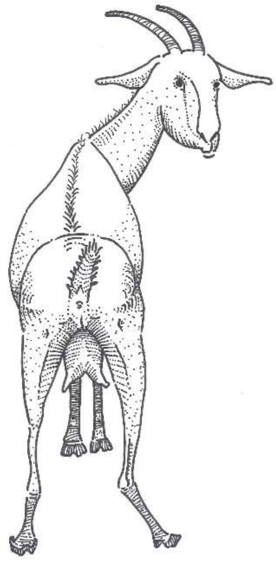	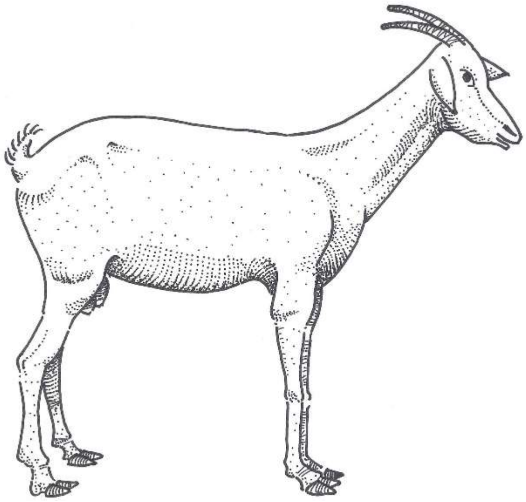
**SCORE 5: Fat and smooth, overweighed animal**<br><br>**Bones of the pelvis and hip:** Difficult to locate, evident fat cover<br>**Tail base:** Evident fat cover<br>**Pelvis:** Markedly convex<br>**Thighs:** Fleshy (rounded, globular)<br>**Lumbar vertebrae:** Line of apophyses invisible (due to fat cover)<br>**Hollow side:** Invisible<br>**Backline:** Invisible (due to fat cover)<br>**Ribs:** Invisible (due to evident fat cover)<br>**Shoulder:** Round shoulder line, evident fat cover<br>**Neckline:** Muscular (globular), hollow of the neck filled	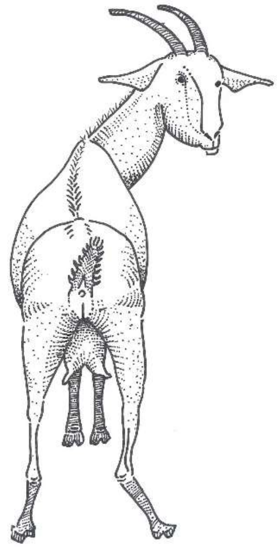	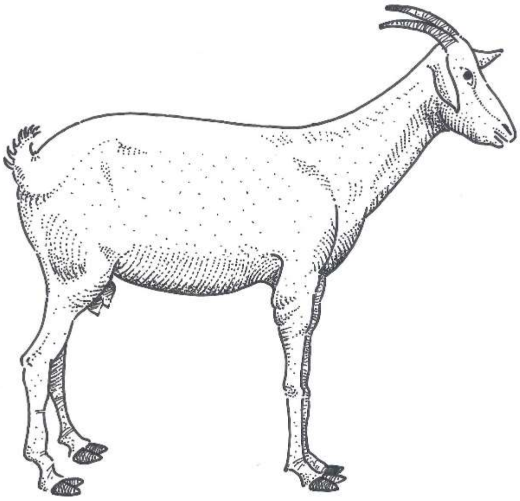

4 REFERENCES

Cisse M., 1995. Une grille de notation de l'état corporel des chèvres Sahéliennes et Djallonké. Fiche technique. ISRA, Dakar, Sénégal, x p.

Cisse M., LY J., Manga R., Boye C., 1994. Use of body condition score for the in vivo estimation of body fat in the Sahel goal. Proc. Soc. Nutr. Physiol., 3: 3.

Cisse M., Mbaye M., Sane I., Korrea A., Ndiaye I., 1992. Seasonal changes in body condition of Senegalese Sahel goat. Relationship with reproduction performances. In: Prod 2d biennial conference of the small ruminant African Reseach Network, AICC, 7-11 December 1992, Arusha, Tanzania : 175-178.

Hervieu J., Schmidely P., Fedele V., Delfa R., 1991. Mesures anatomiques permettant d'expliquer les variations des notes sternales, lombaires et caudales utilisées pour estimer l'état corporel des chèvres laitières. CIHEAM, Options Méditerranéennes, Série séminaire, n°13: 43-56

Imadine M., 1991. Mise au point d'une grille de notation de l'état corporel des caprins du Sahel. Mémoire Dess Productions animales en régions chaudes. Cirad-Emvt, Maisons-Alfort, France, 75 p.

Morand-Fehr P., Branca A., 1987. Méthodes d'estimation de l'état corporel des chèvres reproductrices. In : Proc. Symp. Philostios, L'évaluation des ovins et des caprins méditerranéens, , 22-25 sept. 1987, Fonte Boa, Portugal, p. 182-201.

Vall E., 2020. Guide harmonisé de Notation de l'Etat Corporel (NEC) pour les animaux de ferme du Sahel : Ruminants de grande taille (Bovins, Camelins) et de petite taille (Ovins, Caprins) et équidés (Asins et Equins). CILSS, Cirad, Montpellier, France, 43 p.

5 ANNEXES

Annexe 1. Pictures of goats with BCS varying from 0 to 2

Score	Back view	Right side view
**Score 0: Skeletal animal**	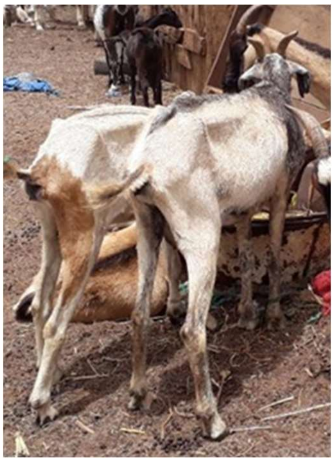	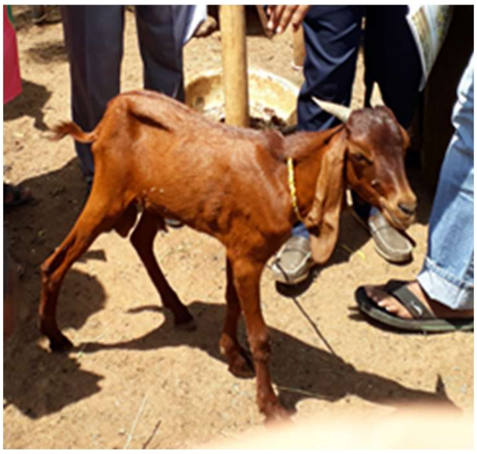
**Score 1: Very thin animal**	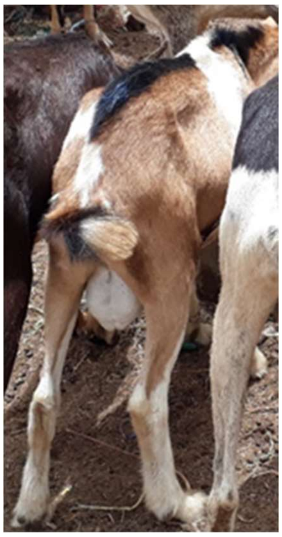	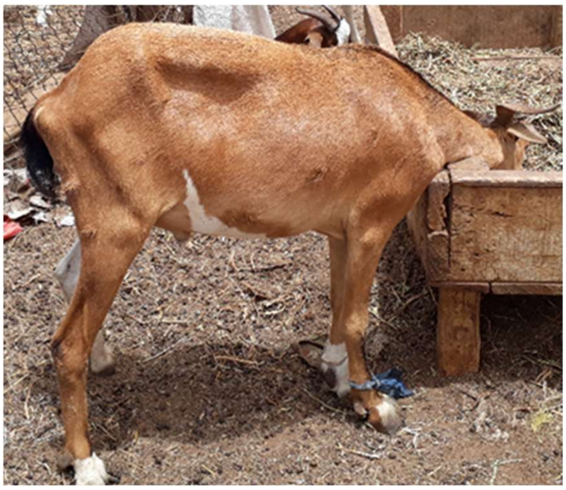
**Score 2: Skinny, lean animal**	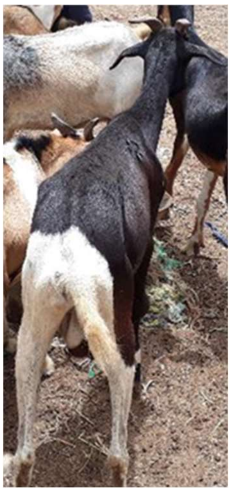	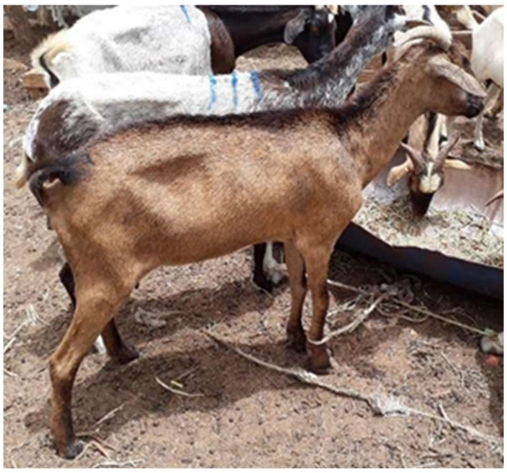

Annexe 2. Pictures of goats with BCS varying from 3 to 5

Score	Back view	Right side view
**Score 3: Animal with a good body condition**	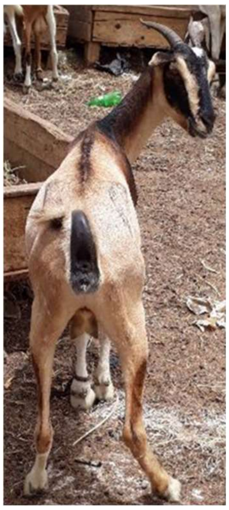	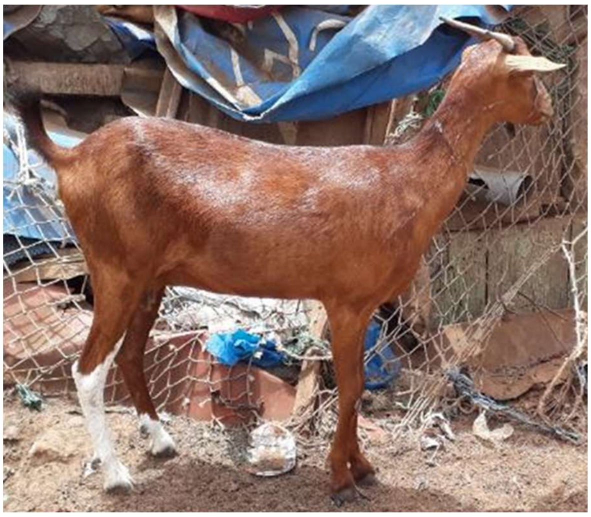
**Score 4: Animal with excellent body condition**	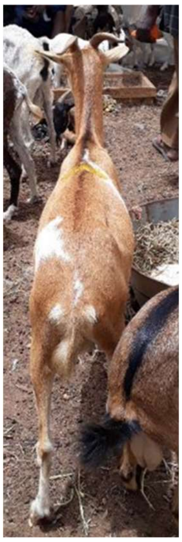	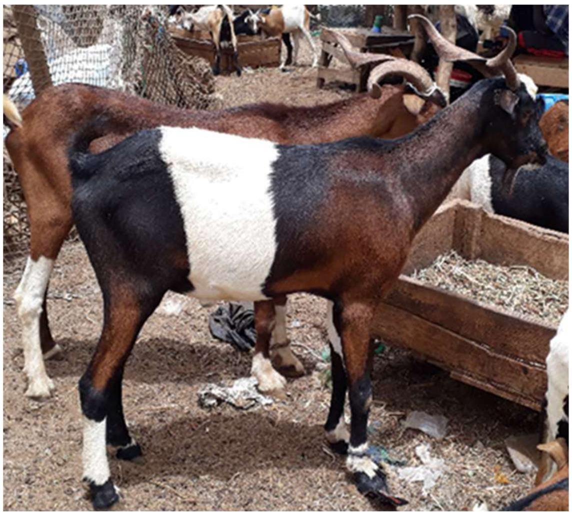
**Score 5: Fat and smooth, overweight animal**	No pictures	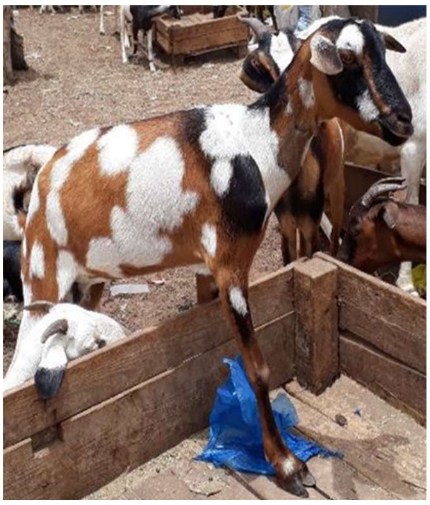
