# Supplementary material for: Standardized body condition scoring system for tropical farm animals (large ruminants, small ruminants, and equines)
Source: Trop Anim Health Prod. 2025 Mar 7;57(2):106. doi: 10.1007/s11250-025-04328-4 (PMC11889029; doi:10.1007/s11250-025-04328-4)
Supplement: Supplementary file 7 — Supplementary file7 (PDF 2497 KB) [file 11250_2025_4328_MOESM7_ESM.pdf]

# Body Condition Scoring Grid for Ndama cattle (Ivory Coast)

Ollo SIB[1], Kréman M. KOUABENAN[2], Eugène KOUADIO[2], et Séverin KOUADJA[2], Eric VALL[3],

(1) CIRAD, UMR SELMET, Bobo-Dioulasso, Burkina Faso.
SELMET, Univ Montpellier, CIRAD, INRAE, Institut Agro, Montpellier, France.
(2) SRPEL, CNRA, Bouaké, Cote d'Ivoire.
(3) CIRAD, UMR SELMET, F-34398 Montpellier, France.
SELMET, Univ Montpellier, CIRAD, INRAE, Institut Agro, Montpellier, France.

October 2023

## Contents

1 INTRODUCTION ..... 2
2 METHODOLOGY ..... 3
2.1 Principles of construction of the BCS grid ..... 3
2.2 Source of BCS grid drawings ..... 4
3 PRESENTATION OF THE BCS GRID ..... 5
3.1 Anatomical landmarks used to rate the BCS ..... 5
3.2 BCS grid for Ndama cattle ..... 6
4 REFERENCES ..... 9
5 ANNEXES ..... 10

# 1 INTRODUCTION

In tropical countries, and especially in Sub-Saharan Africa, subject to significant quantitative and qualitative variations in the availability of fodder during the year, the dissatisfaction of feed needs is a frequent situation for animals reared in grazing systems without a sufficient supplementation with feed concentrates.

The Body Condition Scoring (BCS) is a useful way to assess the state of body reserves (subcutaneous fat, muscle mass) which reflects the animal's production (growth, milk, work) and reproduction capacities. The evaluation of the body reserves of an animal through the scoring of their body condition is important to adjust their diet and appreciate their general state of health, as well as their reproductive and production capacity (meat, milk, work, etc.). Managing body reserves is one way of responding to variability in quality and access to feed resources. In females, reserves play an important buffer role during lactation. They can make up for insufficient intakes from the ration. Indeed, the level of production depends on the nutrients provided by the feeds, but also on the animal's body reserves when the feed-based supplies do not fully cover their needs, especially during the dry season. The BCS impacts the interval between two calving. Overly lean females show a delay in the return of heat after calving, the direct consequence of which is the increase in the parturition interval and consequently a decrease in herd productivity.

The assessment of these reserves through Body Condition Scoring (BCS) represents a management tool for livestock farmers, agricultural advisors, and livestock development stakeholders. BCS is a simple, inexpensive and fast method. Several animals can be scored in one session. It allows to compare the BCS of individuals or herds: 1) raised in different production systems or environments, 2) or, during different seasons (dry season and rainy season). BCS can be used as a tool for monitoring and alerting the nutritional level of domestic animal populations. To do this, BCS alert thresholds and a BCS collection and monitoring system must be defined on reasoned samples of animal populations.

It is an easy-to-use field tool. However, a good mastery of the BCS grid as well as a regular practice of scoring are necessary to obtain precise and reproducible ratings. It can also be used as a tool for monitoring and alerting the nutritional level of animal populations. Thus, the farmer can be called at any time to intervene on the feed ration and / or the health of the animal.

In Sub-Saharan Africa, many studies have been carried out for developing a BCS grid for cattle and especially devoted to Zébu (*Bos Indicus*). For Taurines (*Bos taurus*), well represented in the Sudanian Savannah areas due to their trypano-tolerant nature, there has been little work on scoring their body condition. However, Richard, cited in Le Thiec (1996) and Ezanno (2002), proposed a 5-level BCS grid (Figure 1).

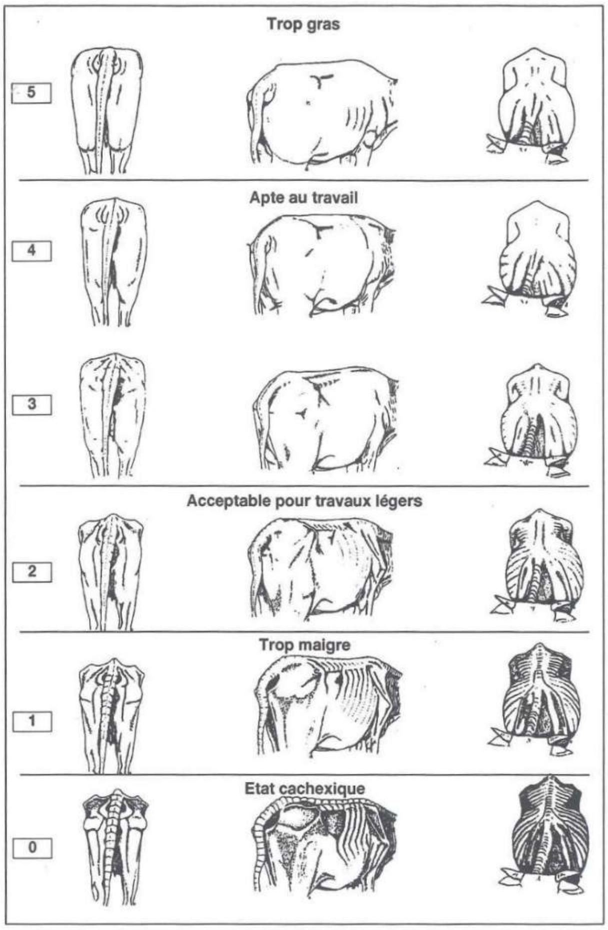


**Figure 1. Système de NEC des bovins Ndama proposé par Richard (cité dans Le Thiec, 1996).**

In 2020, Vall proposed a standardized BCS scoring system for tropical livestock animals for large animals (cattle, camels), small animals (sheep, and goats) and for donkeys, and horses. This document presents this BCS system applied to the Ndama cattle (Bos Taurus) of Ivory Coast.

# 2 METHODOLOGY

## 2.1 Principles of construction of the BCS grid

This document aims to present a BCS grid for Ndama cattle (*Bos Taurus*) of Ivory Coast following the principles and criteria of the harmonized BCS system proposed by Vall (2020) which are as follows:

1) Represent females, because they are the keystone of breeding, because they represent the largest number of breeding animals, and because their body condition is a very good indicator of the good or bad breeding management (in a herd a deterioration of females BCS is indicative of a disorder or problems in rising management);
2) Standardize and simplify the work of assessors by proposing a BCS grid comprising:
    a) A panel of around ten harmonized anatomical points, generally identical for all species, and distributed over three areas of the body: the hindquarters or rump, the thorax and abdomen, the shoulders and the neckline. The detailed description of the anatomical points has been adapted to the case of each species. The ten anatomical landmarks retained are:
        1) Pelvis and hip bone: iliac and ischial spines of the pelvis, and coxo-femoral joint
        2) Base of the tail (caudal strait and caudal vertebrae)

3) Pelvis
4) Thighs
5) Lumbar vertebrae (transverse processes of the lumbar vertebrae)
6) Hollow side
7) Backline (spinous processes of the dorsal vertebrae)
8) Ribs
9) Shoulder: shoulder blade, and humerus joint
10) Neckline (hollow of the neck and general appearance)

b) The same range of scoring (from 0 to 5) for all the species. Beyond 6 levels, the distinction between two BCS levels becomes very subjective and below that it lacks of acuity. The 6-level rating systems are the most widespread in the literature;
c) A “back BCS” and a “right-side BCS”, with a Final BCS corresponding to the average of the two;

## 2.2 Source of BCS grid drawings

As it is not easy to have animals in the same position, we chose to create a BCS grid with drawings representing a typical animal in the different BCS levels and from two perspectives angles (side view, back view). The grid was developed from a series of photos of Ndama cattle taken in Ivory Coast; part of which is presented in the Annex. The drawings were made in Indian ink by Eric Vall.

The photos were taken by Mr Souleymane Kandé from ‘’Laboratoire National d’appui au développement agricole LANADA de Bouaké’ in Bouaké area. The animals were photographed from two perspectives (side view, back view) at approximately 3 m of distance.

# 3 PRESENTATION OF THE BCS GRID

## 3.1 Anatomical landmarks used to rate the BCS

The scoring is made by visual observations of the BCS landmarks distributed on the three parts of the body, represented on the Figure 2 :

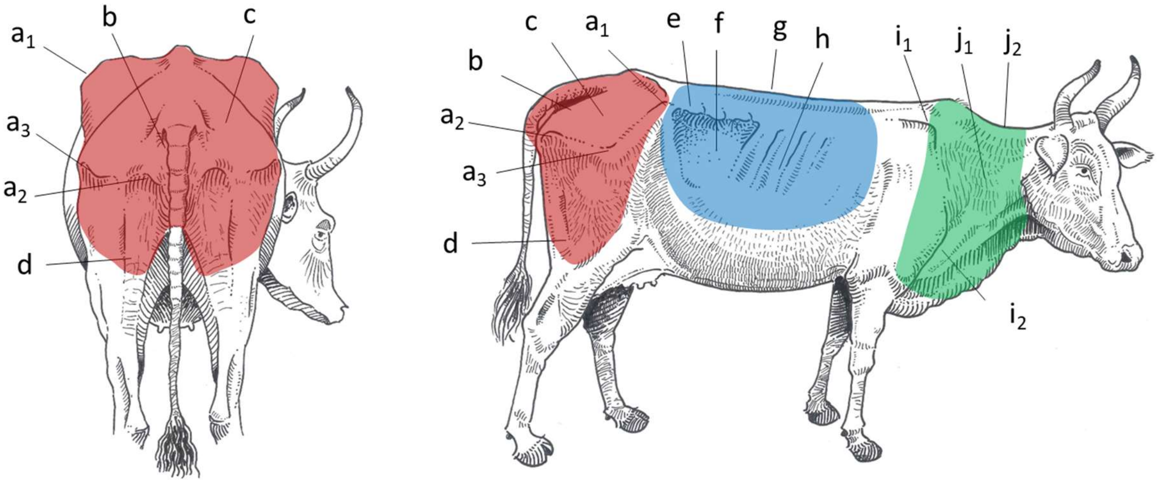


**Hindquarters (rump): Bones of the pelvis and hip:** iliac ($a_1$) and ischial ($a_2$) spines of the pelvis, coxo-femoral joint (a3); **tail base:** tail base and caudal vertebrae (b); **Pelvis:** pelvis cover (c); **Thighs:** thigh covers (d )

**Thorax and abdomen: Lumbar vertebrae:** transverse processes of lumbar vertebrae (e); **Hollow side:** hollow of the side (f); **Backline:** spinous processes of the dorsal vertebrae (g), **Ribs:** ribs appearance (h)

**Shoulder and neck: Shoulder:** shoulder blade ($i_1$), humerus joint ($i_2$); **Neckline:** hollow of the neck ($j_1$), general appearance ($j_2$)

**Figure 2. BCS landmarks for a Ndama cattle (back and right-side views)**

## 3.2 BCS grid for Ndama cattle

**Tableau I. BCS Grid for Ndama cattle**

| | **Hindquarters (rump)** | | | | **Thorax and abdomen** | | | | **Shoulders and neck** | |
|---|---|---|---|---|---|---|---|---|---|---|
| **BCS** | **Bones of the pelvis and hip** | **Tail base** | **Pelvis** | **Thighs** | **Lumbar vertebrae** | **Hollow side** | **Backline** | **Ribs** | **Shoulder** | **Neckline** |
| **0** | Very prominent, skin glued to the bones | Deep caudal strait, Prominent caudal vertebrae | Very concave | Skeletal | Individualized apophyses | Wide and strongly depressed | Very marked over the entire length of the back | All visible, skin glued to the bones | Skeletal shoulder line | Emaciated, hollow of the neck concave |
| **1** | Very prominent | Visible caudal strait and caudal vertebrae | Concave | Very skinny | Line of protruding apophyses marking a sharp angle | Depressed | Marked to the middle of the back | Visible over 75% of the back of the ribcage | Protruding shoulder line | Skinny, hollow of the neck concave |
| **2** | Totally visible | Nascent caudal strait and caudal vertebrae | Flat | Skinny | Line of apophyses with non-sharp angle | Marked | Marked behind the withers | Visible over 50% of the back of the ribcage | Angular shoulder line | Skinny, hollow of the neck concave to filled |
| **3** | Visible, fat deposit sensitive to the touch | Barely visible caudal strait | Flat to convex | Fleshy (dry) | Barely visible line of apophyses with rounded angle | Nascent | Perceptible behind the withers | Visible over 10% of the back of the ribcage | Round shoulder line | Full, hollow of the neck filled |
| **4** | Barely visible, fat cover visible | Filled caudal strait, emerging fat cover | Convex | Fleshy (full) | Line of apophyses detectable | Barely visible | Barely visible | Barely visible, light fat cover | Round shoulder line, emerging fat cover | Muscular, hollow of the neck filled |
| **5** | Difficult to locate, evident fat cover | Invisible caudal strait, evident fat cover | Markedly convex | Fleshy (rounded, globular) | Line of apophyses invisible (due to fat cover) | Barely invisible | Invisible (due to fat cover) | Invisible (due to fat cover) | Round shoulder line, evident fat cover | Muscular (globular), hollow of the neck filled |

## Figure 3. BCS grid for Ndama cattle (commented drawings): scoring from 0 to 2

| Score | Back view | Right side view |
|---|---|---|
| **SCORE 0: Very emaciated, skeletal animal**<br><br>**Bones of the pelvis and hip:** Very prominent, skin glued to the bones<br>**Tail base:** Deep caudal strait, Prominent caudal vertebrae<br>**Pelvis:** Very concave<br>**Thighs:** Skeletal<br>**Lumbar vertebrae:** Individualized apophyses<br>**Hollow flank:** Wide and strongly depressed<br>**Backline:** Very marked over the entire length of the back<br>**Ribs**: All visible, skin glued to the bones<br>**Shoulder:** Skeletal shoulder line<br>**Neckline:** Emaciated, hollow of the neck concave | 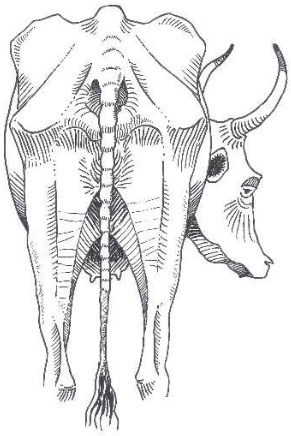 | 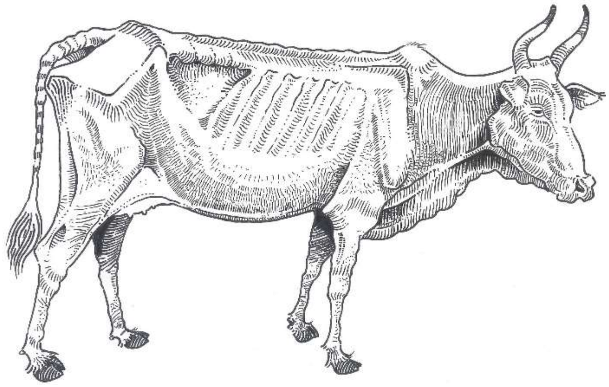 |
| **SCORE 1: Very thin animal**<br><br>**Bones of the pelvis and hip:** Very prominent<br>**Tail base**: Visible caudal strait and caudal vertebrae<br>**Pelvis:** Concave<br>**Thighs**: Very skinny<br>**Lumbar vertebrae:** Line of protruding apophyses marking a sharp angle<br>**Hollow side:** Depressed<br>**Backline:** Marked to the middle of the back<br>**Ribs:** Visible over 75% of the back of the ribcage<br>**Shoulder:** Protruding shoulder line<br>**Neckline:** Skinny, hollow of the neck concave | 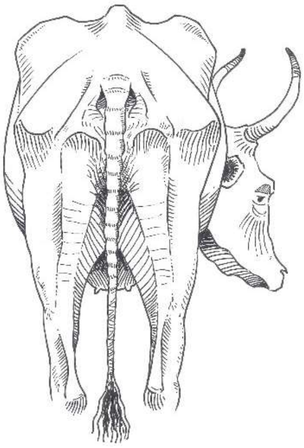 | 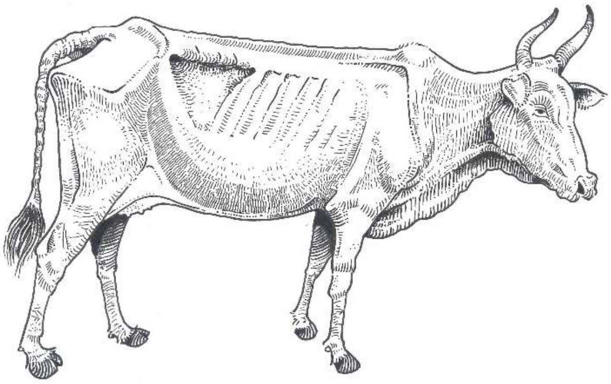 |
| **SCORE 2: Skinny, lean animal**<br><br>**Bones of the pelvis and hip:** Totally visible<br>**Tail base**: Nascent caudal strait and caudal vertebrae<br>**Pelvis:** Flat<br>**Thighs:** Skinny<br>**Lumbar vertebrae:** Line of apophyses with non-sharp angle<br>**Hollow side:** Marked<br>**Backline:** Marked behind the withers<br>**Ribs**: Visible over 50% of the back of the ribcage<br>**Shoulder:** Angular shoulder line<br>**Neckline:** Skinny, hollow of the neck concave to filled | 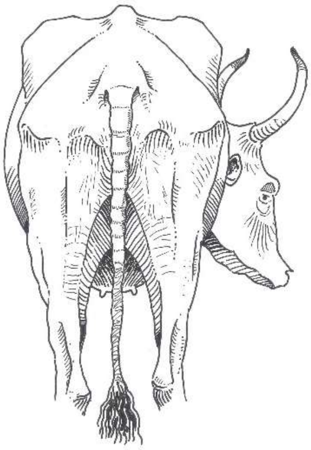 | 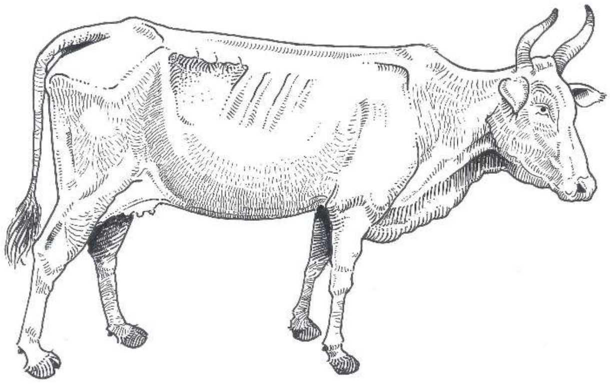 |

## Figure 4 : BCS grid for Ndama cattle (commented drawings): scoring from 3 to 5

| Score | Back view | Right side view |
|---|---|---|
| **SCORE 3: Animal with good body condition**<br><br>**Bones of the pelvis and hip:** Visible, fat deposit sensitive to the touch<br>**Tail base:** Barely visible caudal strait<br>**Pelvis:** Flat to convex<br>**Thighs:** Fleshy (dry)<br>**Lumbar vertebrae:** Barely visible line of apophyses with rounded angle<br>**Hollow side:** Nascent<br>**Backline:** Perceptible behind the withers<br>**Ribs:** Visible over 10% of the back of the ribcage<br>**Shoulder:** Round shoulder line<br>**Neckline:** Full, hollow of the neck filled | 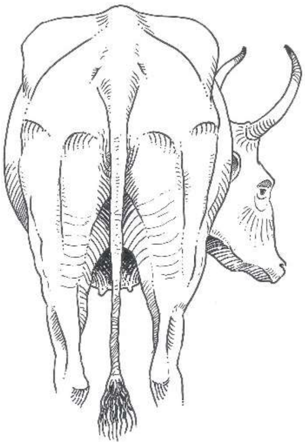 | 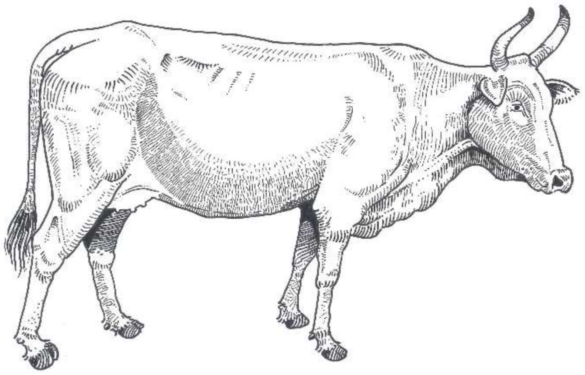 |
| **SCORE 4: Animal with excellent body condition**<br><br>**Bones of the pelvis and hip:** Barely visible, fat cover visible<br>**Tail base:** Filled caudal strait, emerging fat cover<br>**Pelvis:** Convex<br>**Thighs:** Fleshy (full)<br>**Lumbar vertebrae:** Line of apophyses detectable<br>**Hollow side:** Barely visible<br>**Backline:** Barely visible<br>**Ribs**: Barely visible, light fat cover<br>**Shoulder:** Round shoulder line, emerging fat cover<br>**Neckline:** Muscular, hollow of the neck filled | 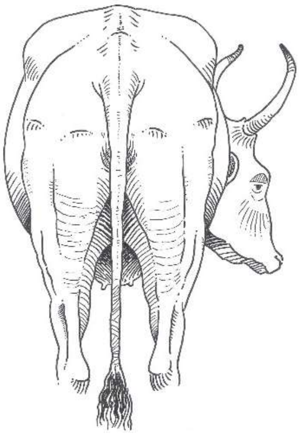 | 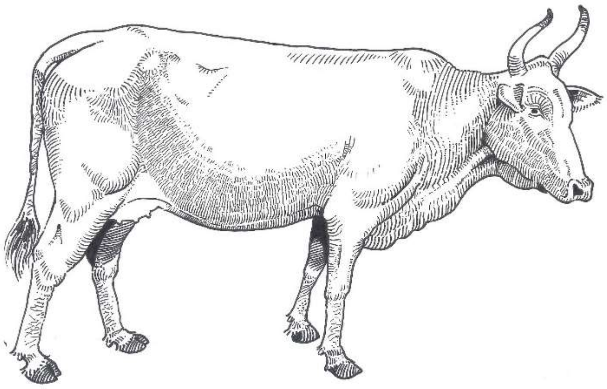 |
| **SCORE 5: Fat and smooth, overweighed animal**<br><br>**Bones of the pelvis and hip:** Difficult to locate, evident fat cover<br>**Tail base**: Invisible caudal strait, evident fat cover<br>**Pelvis:** Markedly convex<br>**Thighs:** Fleshy (rounded, globular)<br>**Lumbar vertebrae:** Line of apophyses invisible (due to fat cover)<br>**Hollow side:** Barely invisible<br>**Backline:** Invisible (due to fat cover)<br>**Ribs:** Invisible (due to fat cover)<br>**Shoulder:** Round shoulder line, evident fat cover<br>**Neckline:** Muscular (globular), hollow of the neck filled | 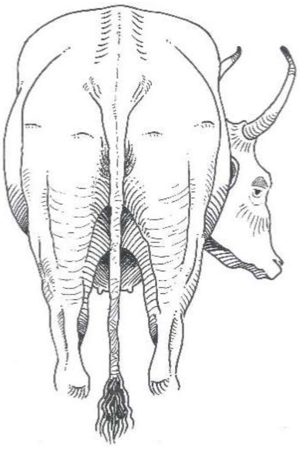 | 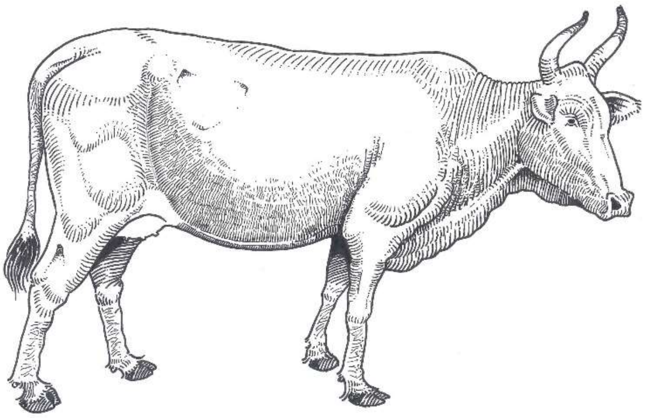 |

# 4 REFERENCES

Ezanno P., 2002. Modélisation de la relation entre l'état des réserves corporelles et la productivité des troupeaux bovins : cas des bovins N'Dama en milieu agro-pastoral extensif de zone tropicale humide (Kolda, Sénégal). Thèse en Biologie de l'Évolution et Écologie, ENSAM, Montpellier, France, x p.

Le Thiec G. (COORD.), 1996. Agriculture africaine et traction animale. CIRAD-SAR, Collection Techniques, Montpellier, France, 438 p.

Vall E., 2020. Guide standardisé pour l'appréciation des réserves corporelles des animaux (bovins, ovins, caprins, camelins, asins et équins) à travers la Note d'Etat Corporel. Document technique, PRAPS, CILSS, Ouagadougou (Burkina Faso), 43 p.

# 5 ANNEXES

**Annexe 1. Pictures of Ndama cattle with BCS varying from 0 to 2**

| Score | Back view | Right side view |
|---|---|---|
| **SCORE 0: Very emaciated, skeletal animal** | 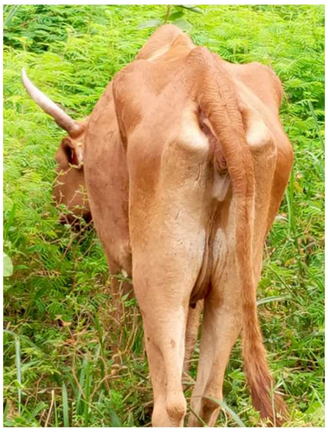 | 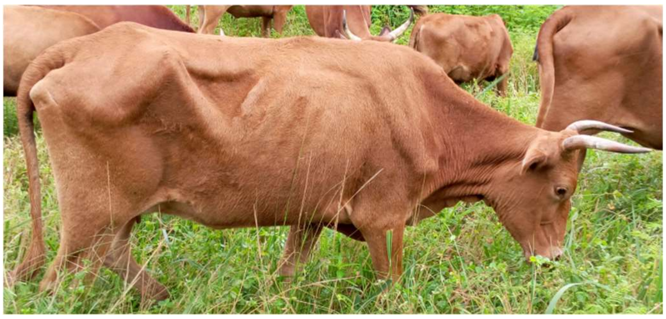 |
| **SCORE 1: Very thin animal** | 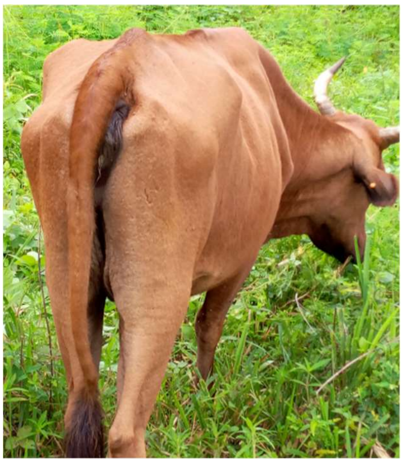 | 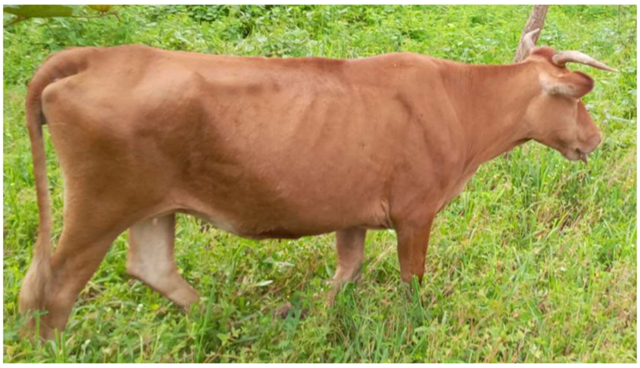 |
| **SCORE 2: Skinny, lean animal** | 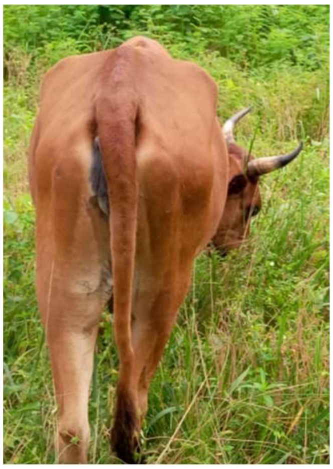 | 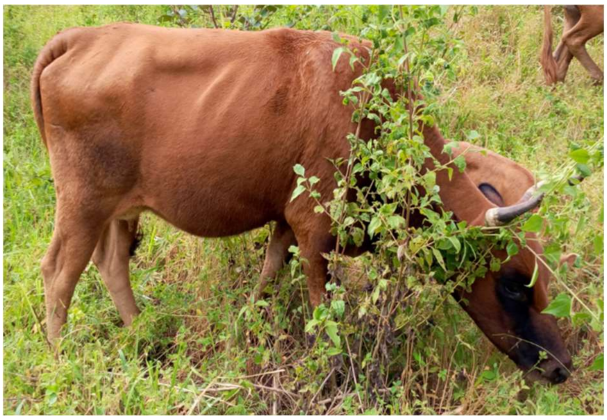 |

## Annexe 2. Pictures of Ndama cattle with BCS varying from 3 to 5

| Score | Back view | Right side view |
|---|---|---|
| **SCORE 3: Animal with good body condition** | 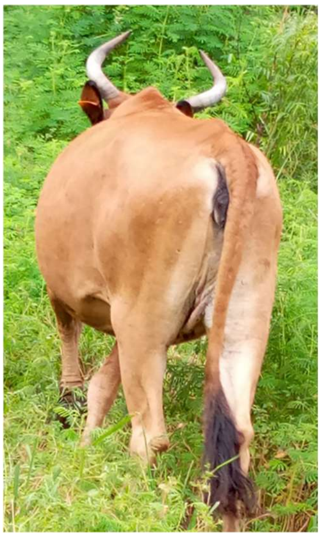 | 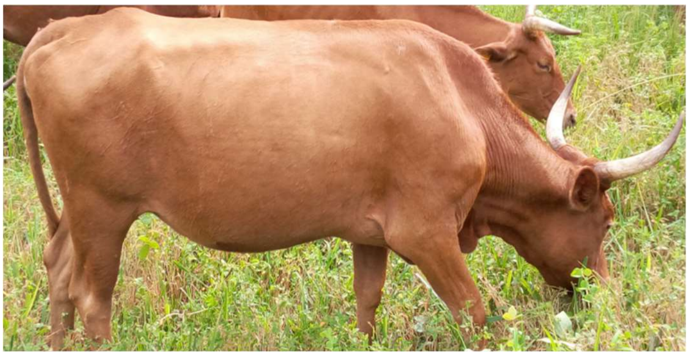 |
| **SCORE 4: Animal with excellent body condition** | 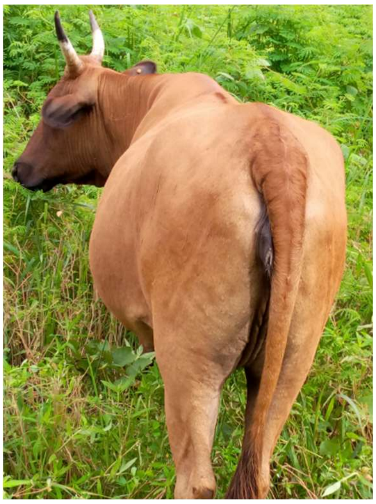 | 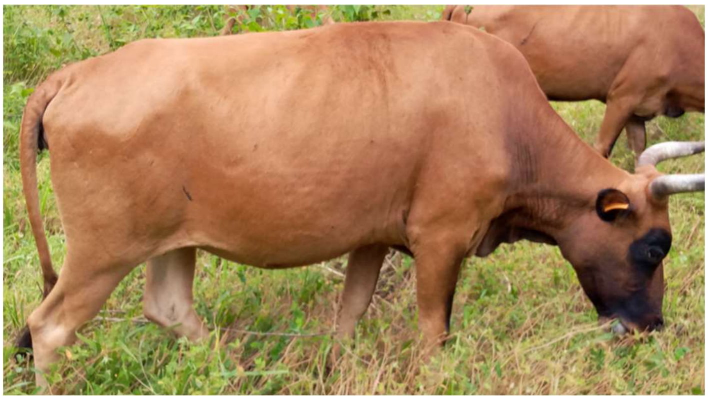 |
| **SCORE 5: Fat and smooth, overweighed animal** | 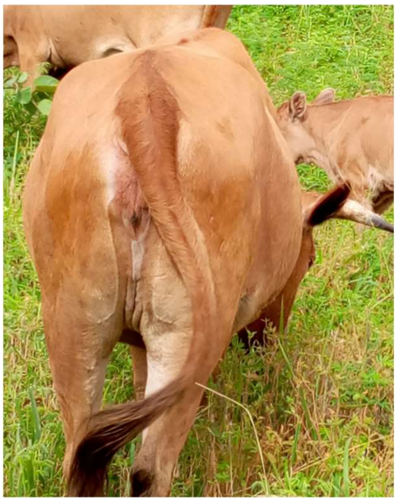 | 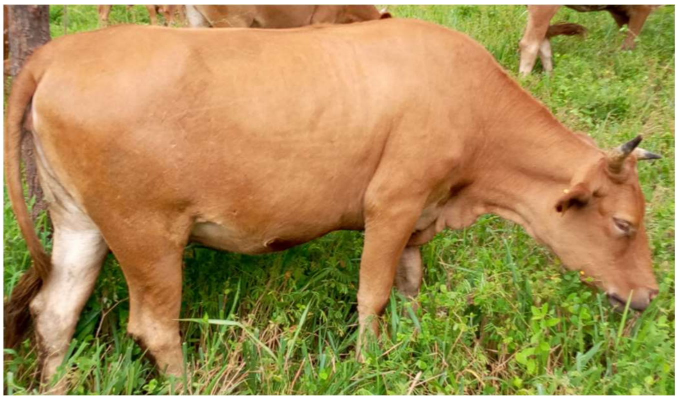 |
